# Astrocytes gate Hebbian synaptic plasticity in the striatum

**DOI:** 10.1038/ncomms13845

**Published:** 2016-12-20

**Authors:** Silvana Valtcheva, Laurent Venance

**Affiliations:** 1 Dynamics and Pathophysiology of Neuronal Networks Team, Center for Interdisciplinary Research in Biology, College de France, CNRS UMR7241/INSERM U1050, MemoLife Labex, 75005 Paris, France; 2 Pierre et Marie Curie University, ED 158, 75005 Paris, France

## Abstract

Astrocytes, via excitatory amino-acid transporter type-2 (EAAT2), are the major sink for released glutamate and contribute to set the strength and timing of synaptic inputs. The conditions required for the emergence of Hebbian plasticity from distributed neural activity remain elusive. Here, we investigate the role of EAAT2 in the expression of a major physiologically relevant form of Hebbian learning, spike timing-dependent plasticity (STDP). We find that a transient blockade of EAAT2 disrupts the temporal contingency required for Hebbian synaptic plasticity. Indeed, STDP is replaced by aberrant non-timing-dependent plasticity occurring for uncorrelated events. Conversely, EAAT2 overexpression impairs the detection of correlated activity and precludes STDP expression. Our findings demonstrate that EAAT2 sets the appropriate glutamate dynamics for the optimal temporal contingency between pre- and postsynaptic activity required for STDP emergence, and highlight the role of astrocytes as gatekeepers for Hebbian synaptic plasticity.

## Abstract (web summary)

Astrocytes regulate synaptic signalling via EAAT glutamate uptake, though whether they play a role in Hebbian plasticity is unknown. Here, the authors find targeting EAAT2 disrupts the emergence of spike timing-dependent plasticity, which highlights the role of astrocytes as gatekeepers for Hebbian plasticity.

## Graphical abstract

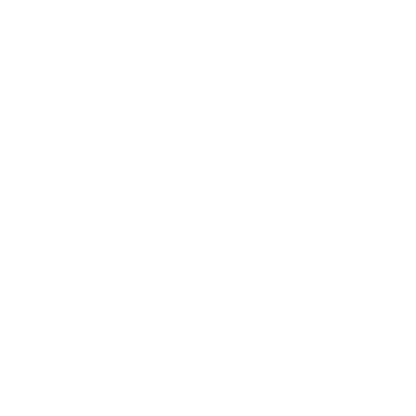

Fast excitatory transmission at central synapses is dependent on glutamate dynamics. Astrocytes play a major role in the precise regulation of glutamate concentration in the extracellular fluid, via their high-affinity glutamate transporters (excitatory amino acid transporters, EAATs), which determine the extent of receptor stimulation by terminating the neurotransmitter signal^1^,^2^,^3^,^4^. Among the five subtypes of EAATs, the largest proportion of glutamate uptake (95%) in the adult forebrain is mediated by the astrocytic EAAT2 (refs 5, 6, 7, 8). Specific deletion of EAAT2 in astrocytes (which express 90% of total EAAT2) revealed that astrocytic EAAT2 contributes to most of the glutamate uptake and that specific EAAT2 deletion in neurons has to this day unidentified consequences^8^,^9^. Decreased levels of EAAT2 associated with increased ambient glutamate have been observed in neurodegenerative and psychiatric diseases^7^,^10^,^11^ and in chronic exposure to drugs of abuse^12^.

EAAT2 is of crucial importance in the maintenance of low glutamate concentrations and for ensuring a high signal-to-noise ratio in synaptic and extrasynaptic transmission^4^,^13^. Astrocytic glutamate uptake via EAAT2 affects both the fast component of the synaptic glutamate transient and slower components by limiting the spill-out to extrasynaptic receptors and the spillover to neighboring synapses^13^,^14^,^15^. Although, astrocytic glutamate transporters are not overwhelmed on physiological activity^16^, synaptic isolation is never reached^17^. Thus, fast removal of glutamate by astrocytes contributes to set the strength and timing of synaptic inputs by controlling peri- and extrasynaptic receptor activation during neuronal activity^18^.

According to Hebbian theory, neural networks refine their connectivity by patterned firing of action potentials in pre- and postsynaptic neurons^19^. Spike timing-dependent plasticity (STDP) is a synaptic Hebbian learning rule that has been the focus of considerable attention in experimental^19^,^20^ and computational^21^,^22^ neuroscience. STDP relies on the precise order and the millisecond timing of the paired activities on either side of the synapse^19^,^20^. However, the conditions required for the emergence of STDP from distributed neural activity remain unclear.

Temporal coding via STDP may be essential for the role of the striatum in learning of motor sequences in which sensory and motor events are associated in a precise time sequence. Corticostriatal long-term plasticity provides a fundamental mechanism for the function of the basal ganglia in procedural learning^23^,^24^. MSNs act as detectors of distributed patterns of cortical and thalamic activity. Thus, the physiological or pathological regulation of EAAT2 expression should play a major role in information processing in the basal ganglia, which is based on a precise time-coding process. EAAT2 is highly expressed in the striatum^7^ and specific knockout of astrocytic EAAT2 leads to pathological repetitive behaviours due to corticostriatal dysfunction^25^. We have previously shown, by dual astrocyte-neuron recordings, that EAAT2 controls corticostriatal transmission and short-term plasticity, and increases the strength of cortical input filtering by the striatum^26^. Here we questioned the role of astrocytes (via EAAT2) in the control of Hebbian plasticity expression, and, more specifically, corticostriatal STDP. We find that under a transient blockade of EAAT2, a non-Hebbian form of plasticity occurring for uncorrelated events replaces STDP. By contrast, EAAT2 overexpression impairs the detection of correlated pre- and postsynaptic activity by MSNs, resulting in the absence of plasticity. We demonstrate here that astrocytes, via EAAT2, set the appropriate glutamate dynamics for the emergence and the establishment of synaptic Hebbian learning rule, such as STDP.

## Results

### Bidirectional STDP within a narrow temporal window

**Figure 1:**
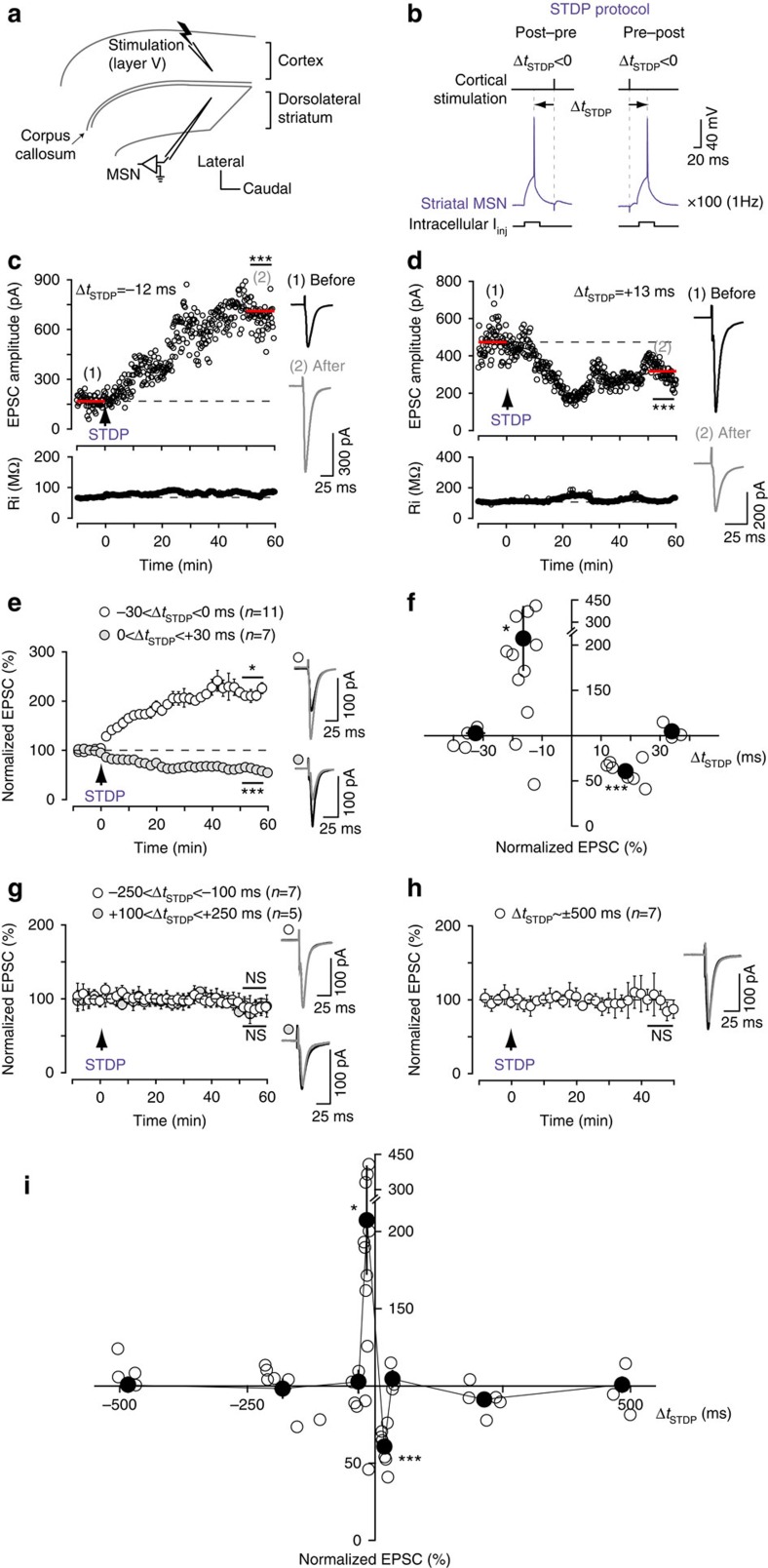
Corticostriatal STDP occurs within a restricted time window. (**a**) Scheme of the recording and stimulating sites in corticostriatal slices. (**b**) STDP pairings: a single spike evoked in the recorded striatal MSN was paired with a single cortical stimulation; this pairing being repeated 100 times at 1 Hz. Δ*t*_STDP_ indicates the time between pre- and postsynaptic stimulations. Δ*t*_STDP_<0 and Δ*t*_STDP_>0 refer to post–pre and pre–post pairings, respectively. (**c**) Example of LTP induced by 100 post–pre pairings (Δ*t*_STDP_=−12 ms). Top, EPSC strength before and after pairings. Bottom, time course of Ri (baseline: 67±0.3 MΩ and 50–60 min after pairings: 79±0.8 MΩ; change of 18%). EPSC traces during 10 min of baseline (1) and at 1h after the STDP protocol (arrow) (2). (**d**) Example of LTD induced by 100 pre–post pairings (Δ*t*_STDP_=+13 ms; Ri, baseline: 106±0.5 MΩ; 50–60 min after pairings: 116±0.5 MΩ; change of 9%). (**e**) Averaged time-course of LTP induced by 100 post–pre pairings and LTD induced by 100 pre–post pairings. (**f**) Bidirectional STDP occurred in a narrow time window: post–pre pairings (−30<Δ*t*_STDP_<0 ms) induced LTP, whereas pre–post pairings (0<Δ*t*_STDP_<+30 ms) induced LTD. Synaptic strength was determined 45–60 min after pairings (empty circles: individual neurons; black circle: average). The *y* axis is discontinuous for clarity; plasticity amplitudes above the interruption are 312 pA, 367 pA and 424 pA. (**g**) Uncorrelated post–pre (−250<Δ*t*_STDP_<−100) and pre–post (+100<Δ*t*_STDP_<+250 ms) pairings induced no significant plasticity. (**h**) Post–pre or pre–post pairings with Δ*t*_STDP_∼±500 ms induced no significant plasticity. (**i**) Graph summarizing STDP occurrence. Bidirectional plasticity was induced over a narrow time window (−30<Δ*t*_STDP_<+30 ms), whereas no plasticity was observed with uncorrelated pairings (−500<Δ*t*_STDP_<−30 ms and +30<Δ*t*_STDP_<+500 ms). Insets correspond to a mean of 60 EPSCs during baseline and at 1 h after STDP pairings. Error bars represent the s.d. (except in **f**,**i**: s.e.m.). *P<0.05; ***P<0.001; NS: not significant by unpaired *t*-test, two-tailed (**c**,**d**) or one sample *t*-test (**e**–**i**).

We investigated the effect of EAAT2 on STDP, using whole-cell recordings from striatal medium-sized spiny neurons (MSNs) in horizontal corticostriatal brain slices from juvenile rats^27^ (Fig. 1a). Baseline excitatory postsynaptic currents (EPSCs) were recorded for 10 min in voltage–clamp mode and then recordings were switched to current–clamp mode to pair a single excitatory postsynaptic potential (EPSP) induced by presynaptic stimulation with a single postsynaptic spike induced by a brief depolarization of the MSN (Fig. 1b). The STDP protocol involved pairing pre- and postsynaptic stimulation with a certain fixed timing interval, Δ*t*_STDP_ (Δ*t*_STDP_<0 indicating that postsynaptic stimulation preceded presynaptic stimulation and Δ*t*_STDP_>0 indicating that presynaptic stimulation preceded postsynaptic stimulation), repeated 100 times at 1 Hz. After the STDP protocol, recordings were obtained in voltage–clamp mode, and EPSCs were monitored for 1 h.

Post- and presynaptic activities paired within a narrow time window (−30<Δ*t*_STDP_<+30 ms) induced bidirectional STDP in MSNs. An example of the timing-dependent long-term potentiation (t-LTP) induced by post–pre pairings (Δ*t*_STDP_=−12 ms) is illustrated in Fig. 1c; the mean baseline EPSC amplitude was 168±5 pA before pairings, and increased by 324% to 711±22 pA 1 h after pairings. Ri remained stable over this period. Conversely, pre–post pairings (Δ*t*_STDP_=+13 ms) induced timing-dependent long-term depression (t-LTD), as shown in the example in Fig. 1d: the mean baseline EPSC amplitude, 474±10 pA, had decreased by 33%, to 318±7 pA, 1 h after pairing. To summarize, post–pre pairings (−30<Δ*t*_STDP_<0 ms) induced t-LTP (mean EPSC amplitude recorded 60 min after protocol induction: 207±35% of baseline, *P*=0.0116, *n*=11; 9 of 11 cells displayed LTP), whereas pre–post pairings (0<Δ*t*_STDP_<+30 ms) induced t-LTD (61±5%, *P*=0.0001, *n*=7; 7/7 cells displayed LTD) (Fig. 1e,f,i), resulting in anti-Hebbian STDP. We have shown that GABA controls the polarity of corticostriatal STDP^28^ and that Hebbian^29^,^30^ or anti-Hebbian^27^,^31^,^32^ STDP were observed, depending on whether GABA_A_ receptor antagonists are used. The pairings for Δ*t*_STDP_∼−30 ms and Δ*t*_STDP_∼+30 ms did not induce plasticity (97±5%, *P*=0.6205, *n*=4 and 105±5%, *P*=0.4670, *n*=3). Less correlated pairings (Δ*t*_STDP_<−30 ms and Δ*t*_STDP_>+30 ms) failed to induce long-term synaptic efficacy changes. Indeed, for −250<Δ*t*_STDP_<−100 ms and +100<Δ*t*_STDP_<+250 ms, we observed no plasticity (98±6%, *P*=0.7931, *n*=7 and 91±4%, *P*=0.1067, *n*=5, respectively; Fig. 1g,i). Uncorrelated pairings up to ±500 ms, the maximum interval between the postsynaptic action potential and the presynaptic stimulation paired at 1 Hz, also failed to induce long-term synaptic efficacy changes (103±5%, *P*=0.4577, *n*=7; Fig. 1h,i). Thus, post- and presynaptic activities paired only within a narrow temporal window, spanning 60 ms (−30<Δ*t*_STDP_<+30 ms), efficiently induce bidirectional STDP (Fig. 1i).

### EAAT2 gates the polarity and temporal window of STDP

**Figure 2:**
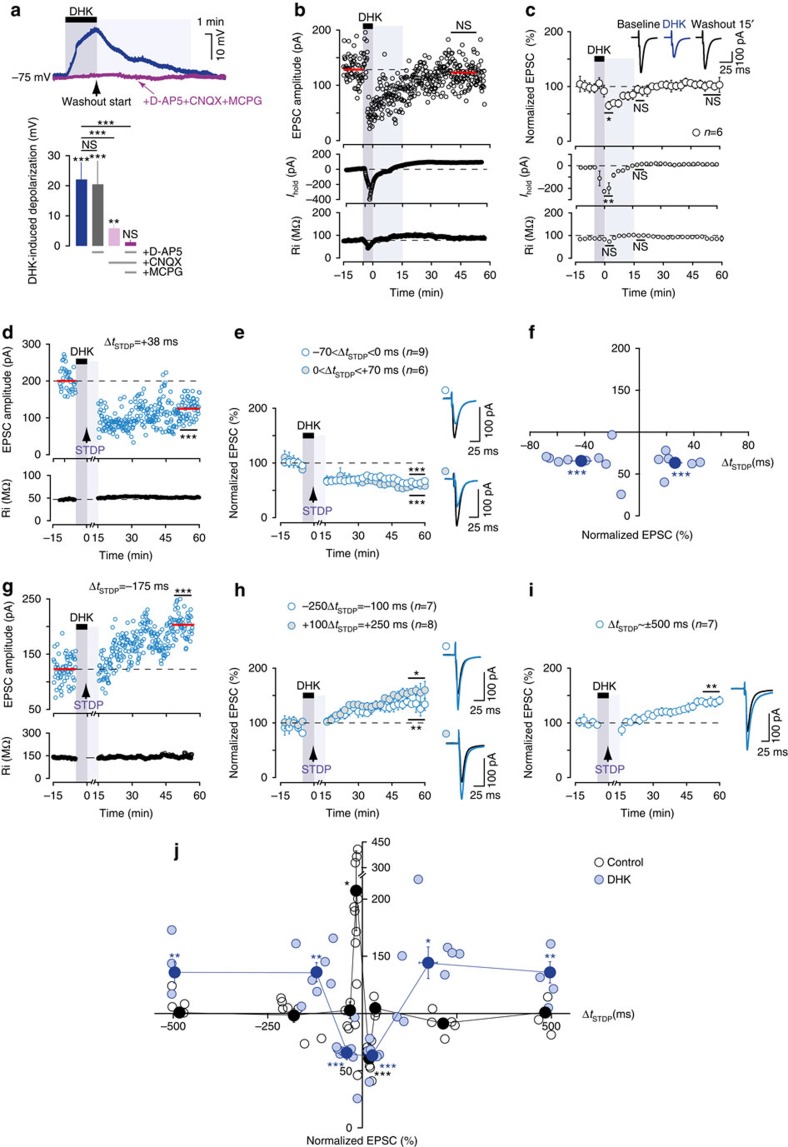
EAAT2 activity gates STDP polarity and time window. (**a**) Current–clamp recording of MSN in the absence of cortical stimulation showing that brief DHK application (300 μM for 5 min) induced significant depolarization, indicating the presence of ambient glutamate in the slice. This depolarization was fully reversed after 15 min of DHK washout and was dependent on AMPAR and type-I/II mGluR, but not NMDAR. (**b**,**c**) DHK application had no effect on long-term synaptic efficacy changes estimated from 15 min after DHK washout (example in **b** and averaged time-course of experiments in **c**). The brief application of DHK without the STDP protocol induced a transient decrease in EPSC amplitude and an inward shift in *I*_holding_ (light gray area). Both EPSC amplitude and *I*_holding_ had fully recovered 15 min after DHK washout. Ri remained unchanged during and after DHK application. The effects of DHK were fully reversible and, thus, compatible with the estimation of long-term synaptic efficacy changes. (**d**) Example of LTD induced by 100 pre–post pairings (Δ*t*_STDP_=+38 ms) with a transient blockade of EAAT2 by DHK (300 μM for 5 min, dark gray area; the light gray area indicates DHK washout). Top, EPSC strength before and after pairings. Bottom, time course of Ri (baseline, 47±0.2 MΩ; 50–60 min after pairings, 51±0.1 MΩ; change of 10%). (**e**) Averaged time-course of experiments with the transient blockade of EAAT2 with DHK, showing the induction of LTD for both post–pre (−70<Δ*t*_STDP_<0 ms) and pre–post (0<Δ*t*_STDP_<+70 ms) pairings. (**f**) LTD expression for −70<Δ*t*_STDP_<+70 ms with DHK. Synaptic strength was assessed 45–60 min after pairings (light blue circles: individual neurons; dark blue circle: average). (**g**) Example of LTP induced by 100 post–pre pairings (Δ*t*_STDP_=−175 ms) during the transient blockade of EAAT2 with DHK (Ri, baseline: 136±0.5 MΩ; 50–60 min after pairings: 145±1 MΩ; change of 6%). (**h**) Averaged time-course of experiments with transient EAAT2 blockade with DHK during pairings, inducing LTP for both post–pre (−250<Δ*t*_STDP_<−100 ms) and pre–post (+100<Δ*t*_STDP_<+250 ms) pairings. (**i**) Averaged time-course of experiments with transient EAAT2 blockade with DHK during pairings, inducing LTP for Δ*t*_STDP_∼±500 ms. (**j**) Time window for long-term synaptic strength for post–pre and pre–post pairings (−500<Δ*t*_STDP_<+500 ms) in control conditions and in the presence of DHK. In controls, bidirectional plasticity was induced over a narrow time window (−30<Δ*t*_STDP_<+30 ms) and no plasticity was observed with uncorrelated pairings (−500<Δ*t*_STDP_<−30 ms and +30<Δ*t*_STDP_<+500 ms). During transient EAAT2 blockade, plasticity was observed regardless of the Δ*t*_STDP_ value: LTD for narrow Δ*t*_STDP_ (−70<Δ*t*_STDP_<+70) and LTP for a larger Δ*t*_STDP_ (−500<Δ*t*_STDP_<−70 ms and +70<Δ*t*_STDP_<+500 ms). Insets correspond to the mean of 60 EPSCs during baseline and at 1 h after STDP pairings. Error bars represent the s.d. (except in panel **a**,**f**,**j**: s.e.m.) *P<0.05; **P<0.01; ***P<0.001; NS: not significant by unpaired *t*-test, two-tailed, inside groups after one-way ANOVA; *post hoc* Bonferroni comparisons test (**a**), unpaired *t*-test, two-tailed (**b**,**d**,**g**) or one sample *t*-test (**c**,**e**,**f**,**h**,**i**,**j**).

Investigation of the role of astrocytic glutamate uptake in corticostriatal STDP required the transient blocking of EAAT2 during the STDP pairings (see Methods section). We considered a pharmacological approach to be most appropriate for this purpose. We previously showed, by dual astrocyte-neuron recordings, that dihydrokainate (DHK; 300 μM), a selective non-transportable inhibitor of EAAT2 (ref. 33), efficiently blocked most of the transporter-mediated currents in striatal astrocytes on corticostriatal stimulation^26^. Brief EAAT2 blockade with DHK for 5 min resulted in a marked depolarization of the recorded MSN in current–clamp mode in the absence of cortical stimulation (22±2 mV, *P*<0.0001, *n*=14; Fig. 2a). This effect was fully reversible after 15 min of DHK washout. These findings suggest that the slice contained sufficiently large amounts of glutamate to induce postsynaptic depolarization during EAAT2 blockade. DHK-induced depolarization involved AMPAR and type-I/II mGluR activation (Fig. 2a). Indeed, during the concomitant inhibition of AMPAR with CNQX (20 μM) and of type-I/II mGluR with MCPG (500 μM) no significant depolarization was observed (1.0±0.3 mV, *P*=0.5872, *n*=7). NMDAR inhibition with D-AP5 (50 μM) did not prevent DHK-induced depolarization (one-way analysis of variance (ANOVA): *P*<0.0001; *post hoc* Bonferroni-corrected pairwise comparisons: DHK-D-AP5: *P*>0.05, DHK-CNQX: *P*<0.001, DHK-D-AP5+CNQX+MCPG: *P*<0.001; Fig. 2a).

We then ensured that brief (5 min) EAAT2 blockade induced no long-term change in synaptic efficacy. A stable baseline was established over a period of 10 min. We then applied DHK for 5 min without STDP pairing. As exemplified in Fig. 2b,c, we observed a transient decrease in EPSC amplitude (65±9%, *P*=0.0105, *n*=6) due to AMPAR desensitization, as previously reported^26^, and an inward shift of *I*_holding_ (−199±41 pA, *P*=0.0022; Ri was not significantly affected, *P*=0.8182; Fig. 2c). These effects were fully reversed 15 min after DHK removal (93±9%, *P*=0.4749 and 11±15 pA, *P*=0.1797, respectively; Fig. 2c). Thus, transient EAAT2 blockade with DHK was compatible with the estimation of long-term changes in synaptic efficacy.

For transient EAAT2 blockade during STDP pairings, we observed a profound change in STDP, as synaptic plasticity extended over the entire temporal window: LTD for a narrow Δ*t*_STDP_ (−70<Δ*t*_STDP_<+70 ms) and LTP for a broader Δ*t*_STDP_ (−250<Δ*t*_STDP_<−100 ms, +100<Δ*t*_STDP_<+250 ms and Δ*t*_STD_=±500 ms) (Fig. 2). An example of LTD induced by post–pre pairings (Δ*t*_STDP_=+38 ms) under transient EAAT2 blockade with DHK (300 μM) is shown in Fig. 2d; the mean baseline EPSC amplitude was 200±5 pA before pairings and had decreased by 38%, to 125±3 pA, 1 h after pairings. Both post–pre and pre–post pairings induced LTD in a Δ*t*_STDP_ spanning 140 ms (−70<Δ*t*_STDP_<+70 ms) (66±6%, *P*=0.0005, *n*=9; 8/9 cells displayed LTD for −70<Δ*t*_STDP_<0 ms and 63±5%, *P*=0.0008, *n=*6; 6/6 cells displayed LTD for 0<Δ*t*_STDP_<+70 ms; Fig. 2e,f). LTD was of similar amplitude for post–pre and pre–post pairings (*P*=0.7924). For more uncorrelated pairings (Δ*t*_STDP_<−70 ms and Δ*t*_STDP_>+70 ms), LTP extended over the entire temporal window until ±500 ms. Indeed, as exemplified in Fig. 2g, we observed LTP for post-pairing with a Δ*t*_STDP_=−175 ms under transient EAAT2 blockade (mean baseline EPSC amplitude of 123±3 pA before pairings, increasing by 66%, to 203±3 pA, 1 h after pairings). In summary, we observed LTP for −250<Δ*t*_STDP_<−70 ms and +70<Δ*t*_STDP_<+250 ms (136±8%, *P*=0.0049, *n*=7; 6/7 cells displayed LTP and 144±14%, *P*=0.0148, *n*=8; 6/8 cells displayed LTP, respectively; Fig. 2h,j). We then assessed plasticity induction for the most uncorrelated Δ*t*_STDP_ that could be achieved with a pairing frequency of 1 Hz (that is, Δ*t*_STDP_=±500 ms), and we observed LTP (136±9%, *P*=0.0085, *n*=7; 6/7 cells displayed LTP; Fig. 2i,j). LTP was of similar amplitude for post–pre and pre–post pairings (*P*=0.6325). We previously showed that bidirectional STDP was equally frequent in MSNs involved in the direct and indirect pathways^28^. Here, the occurrence of plasticity under EAAT2 blockade indicates a lack of segregation between the two trans-striatal pathways.

To confirm these findings, we then used another EAAT2 inhibitor, WAY-213,613, structurally distinct from DHK. Both DHK and WAY-213,613 are non-substrate competitive inhibitors (non-transported) of glutamate uptake^33^,^34^. We ensured that transient EAAT2 blockade with WAY-213,613 was reversible and, thus, compatible with the estimation of long-term changes in synaptic efficacy. The bath application of WAY-213,613 (50 μM) for 5 min induced a transient, non-significant decrease in EPSC amplitude (with no change in Ri). This effect was fully reversible within 5 min (*n*=6; Supplementary Fig. 1a–c). For transient EAAT2 blockade with WAY-213,613 (50–100 μM) during STDP pairings (for −70<Δ*t*_STDP_<+70 ms and for Δ*t*_STDP_=±200 ms), we observed a profound modification of STDP (similar to that observed with DHK): LTD or no plasticity for a narrow Δ*t*_STDP_ (−70<Δ*t*_STDP_<+70 ms) and LTP for a broader Δ*t*_STDP_ (Δ*t*_STDP_=±200 ms; Supplementary Fig. 1d–i). First, for −70<Δ*t*_STDP_<+70 ms with WAY-213,613 (50 μM), no plasticity was observed, as exemplified in the Supplementary Fig. 1d. Both post–pre and pre–post pairings (−70<Δ*t*_STDP_<+70 ms) failed to induce significant plasticity (104±5%, *P*=0.4600, *n*=5; 1/5 cells displayed LTD; Supplementary Fig. 1e). With 100 μM WAY-213,613, the incidence of LTD was higher, as exemplified in the Supplementary Fig. 1f, even though, in average no significant LTD was induced for pairings at −70<Δ*t*_STDP_<+70 ms (80±11%, *P*=0.1061, *n*=8; 5/8 cells showed LTD; Supplementary Fig. 1g). LTP was observed for uncorrelated pairings (Δ*t*_STDP_=±200 ms). An example of LTP induced by post–pre pairings (Δ*t*_STDP_=−200 ms) during the transient blockade of EAAT2 with WAY-213,613 (50 μM) is shown in the Supplementary Fig. 1h. In summary, we observed LTP for Δ*t*_STDP_=±200 ms (166±21%, *P*=0.0150, *n*=8; 7/8 cells displayed LTP; Supplementary Fig. 1i).

Thus, during the transient blockade of EAAT2 with either DHK or WAY-213,613, any paired activity on either side of the synapse, regardless of Δ*t*_STDP_, was able to modify synaptic efficacy in the long term (Fig. 2j). This finding contrasts strongly with the STDP observed in control conditions, in which EAAT2 activity was unaffected. In conclusion, the correct functioning of EAAT2 allows the expression of a bidirectional order-dependent STDP during a restricted time window.

### EAA2 blockade-induced depolarization and plasticity

**Figure 3:**
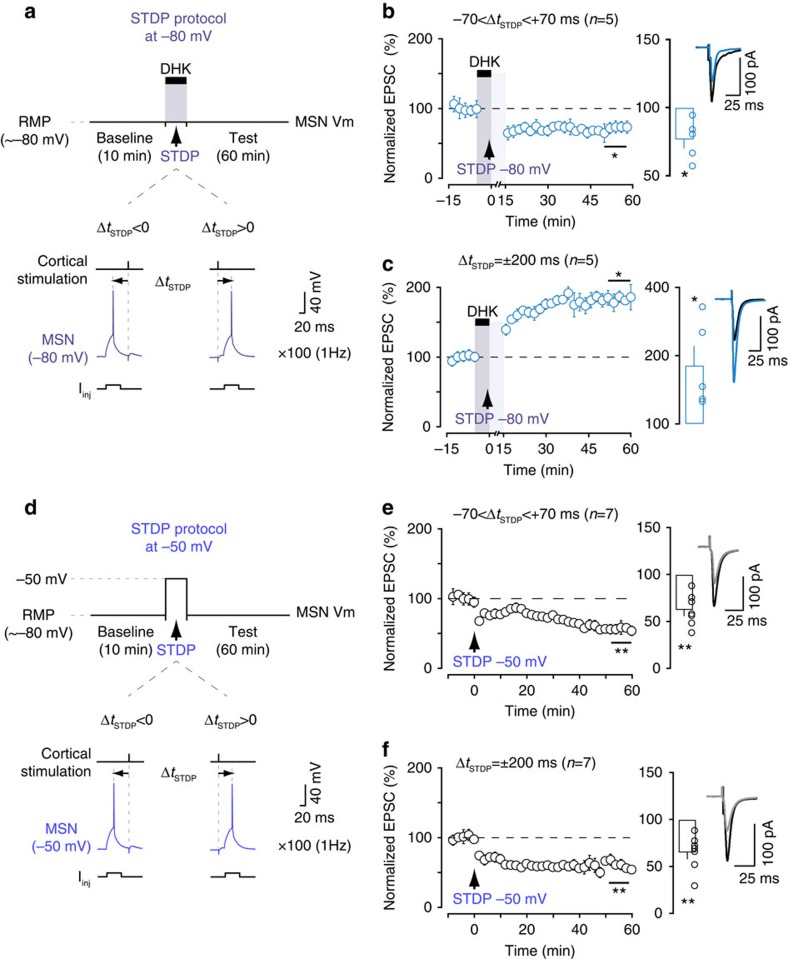
Postsynaptic depolarization cannot account for plasticity under EAAT2 blockade. (**a**–**c**) Experimental protocol (**a**) and averaged time-course of STDP experiments (**b**,**c**) with the recorded MSN maintained at −80 mV by intracellular current injection during the STDP pairings. LTD and LTP were induced with pairings at −70<Δ*t*_STDP_<+70 ms (**b**) and Δ*t*_STDP_=±200 ms (**c**), respectively. The prevention of DHK-induced depolarization did not impair the plasticity observed when MSN was depolarized. (**d**–**f**) Experimental protocol and summary of STDP experiments in which the recorded MSN was held at −50 mV (**d**), performed with pairings at −70<Δ*t*_STDP_<+70 ms (**e**) and Δ*t*_STDP_=±200 ms (**f**), respectively; in these conditions, only LTD was observed. Insets correspond to the average of 60 EPSCs during baseline and at 1 h after STDP pairings. Error bars represent the s.d. (except in bar graphs: s.e.m.). *P<0.05; **P<0.01 by one sample *t*-test (**b**,**c**,**e**,**f**).

We investigated whether the observed plasticity was due to the transient depolarization induced by EAAT2 blockade. For this purpose, we maintained the recorded MSNs at −80 mV by intracellular current injection (close to MSN resting membrane potential) during STDP pairings, to prevent DHK-induced depolarization (Fig. 3a). In these conditions, pairings for −70<Δ*t*_STDP_<+70 ms and Δ*t*_STDP_=±200 ms induced LTD (77±7%, *P*=0.0233, *n*=5; 5/5 cells displayed LTD; Fig. 3b) and LTP (186±28%, *P*=0.0382, *n*=5; 5/5 cells displayed LTP; Fig. 3c), respectively. These results are similar to those obtained in presence of DHK when neurons were not maintained at −80 mV (Fig. 2). Thus, the depolarization of the postsynaptic MSN induced by EAAT2 blockade does not account for the observed plasticity.

We then investigated whether postsynaptic depolarization alone (without DHK) during STDP pairings mimicked the effects of transient EAAT2 blockade. When MSNs were held at −50 mV in the absence of DHK during the STDP protocol (Fig. 3d), pairings for −70<Δ*t*_STDP_<+70 ms and for Δ*t*_STDP_=±200 ms induced exclusively LTD (65±7%, *P*=0.0029, *n*=7, 7/7 cells displayed LTD and 62±6%, *P*=0.0011, *n*=7, 7/7 cells displayed LTD, respectively; Fig. 3e,f). This result is in accordance with LTD induced with sustained depolarization in visual cortex^35^, and with hippocampal depolarization-induced LTD^36^. Thus, postsynaptic depolarization in the absence of DHK is not sufficient to reproduce the effects of transient EAAT2 blockade. Glutamate spillover is, therefore, likely to contribute to the observed plasticity.

### GABA-dependent LTD under transient EAAT2 blockade

**Figure 4:**
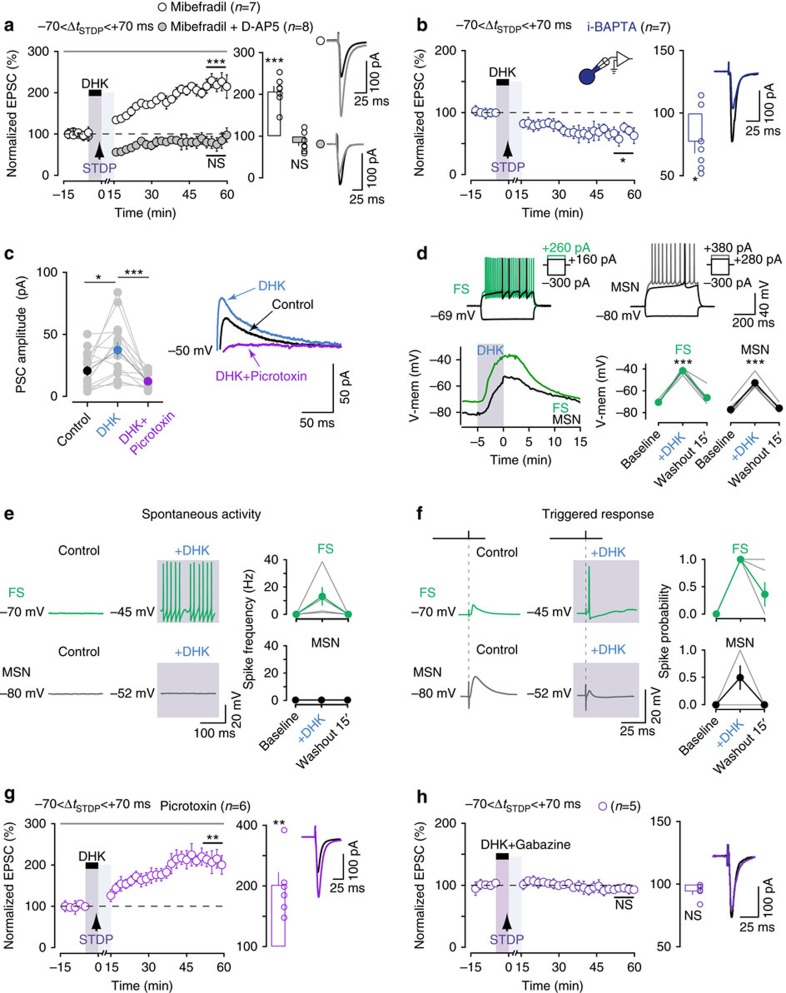
GABA-dependent LTD under transient EAAT2 blockade. (**a**) Blocking L- and T-type VSCCs with mibefradil (20 μM) under transient EAAT2 blockade impaired LTD and revealed potent LTP. This LTP was NMDAR-mediated, because it was prevented by the application of mibefradil (20 μM) together with D-AP5 (50 μM). (**b**) i-BAPTA did not impair the LTD observed under transient EAAT2 blockade. (**c**) Inhibitory currents recorded in MSNs held at −50 mV in control conditions, with DHK and with DHK+picrotoxin (50 μM; *n*=14). (**d**) Top, characteristic voltage responses of one FS cell and one MSN to a series of 500 ms current pulses. Bottom, depolarization of FS cells and MSNs induced by DHK application. Left: example of changes in Vm before, during and after DHK application, in one FS cell and one MSN; right: mean values. (**e**) DHK-induced depolarization led to firing activity in FS cells but not in MSNs. (**f**) Under EAAT2 blockade, cortical stimulation evoked an action potential in all recorded FS cells whereas subthreshold EPSPs were observed in MSNs. (**g**) Picrotoxin (50 μM) prevented the LTD induced by pairings at −70<Δ*t*_STDP_<+70 ms under EAAT2 blockade, and revealed LTP. (**h**) Co-application of gabazine (10 μM) with DHK during STDP pairings prevented the expression of LTD. Insets correspond to the mean of 60 EPSCs during baseline and at 1 h after STDP pairings. Error bars represent the s.d. (**a**,**b**,**g**,**h**) or the s.e.m. (**c**–**f** and bar graphs in panels **a**,**b**,**g**,**h**). *P<0.05; **P<0.01; ***P<0.001; NS: not significant by one sample *t*-test (**a**,**b**,**g**,**h**), one-way repeated-measures ANOVA; *post hoc* Bonferroni-corrected pairwise comparisons (**c**) or paired *t*-test, two-tailed (**d**).

We then investigated the receptors involved in the synaptic plasticity induced under transient EAAT2 blockade. We first investigated the receptors involved in the LTD observed for pairings at −70<Δ*t*_STDP_<+70 ms. In control conditions, corticostriatal t-LTD is mediated by CB_1_R^16^,^17^,^18^. We, therefore, first determined whether the LTD observed under EAAT2 blockade was CB_1_R-mediated. Following the bath application of a CB_1_R-specific antagonist (AM251; 3 μM), LTD was still observed under EAAT2 blockade (69±8%, *P*=0.0019, *n*=11; 10/11 cells showed LTD; Supplementary Fig. 2a), indicating that LTD was not CB_1_R-mediated. mGluRs and NMDARs located outside the synapse can be activated by glutamate spillover promoted by EAAT2 blockade^15^,^37^,^38^,^39^,^40^. We, therefore, investigated the involvement of mGluRs and NMDARs in LTD under EAAT2 blockade for pairings at −70<Δ*t*_STDP_<+70 ms. The inhibition of type I/II mGluRs with MCPG (500 μM) or of NMDARs with D-AP5 (50 μM) had no effect on the establishment of LTD (62±9%, *P*=0.0279, *n*=4; 4/4 cells displayed LTD and 61±5%, *P*=0.0003, *n*=7; 7/7 cells displayed LTD, respectively; Supplementary Fig. 2b). We then examined the involvement of L- and T-type voltage-sensitive calcium channels (VSCCs), which can be activated by DHK-induced depolarization. Under EAAT2 blockade, bath-applied mibefradil (20 μM), a specific antagonist of T-type VSCCs (also blocking L-type VSCCs at concentrations above 18 μM) not only prevented LTD, but also revealed potent LTP (207±13%, *P*=0.0002, *n*=7; 7/7 cells displayed LTP; Fig. 4a). This LTP, unmasked by VSCC inhibition, was mediated by NMDARs, because it was prevented by the co-application of mibefradil and D-AP5 (84±8%, *P*=0.0680, *n*=8; 1/8 cells displayed LTP; Fig. 4a).

Given the involvement of VSCCs in the LTD observed under EAAT2 blockade, we investigated the calcium dependence of LTD at the level of the recorded MSN. To do so, we delivered intracellularly a fast calcium buffer, BAPTA, (i-BAPTA, 10 mM) through the patch-clamp pipette in the recorded MSN. Under EAAT2 blockade, i-BAPTA had no effect on LTD (77±9%, *P*=0.0482, *n*=7; 5/7 cells displayed LTD at −70<Δ*t*_STDP_<+70 ms; Fig. 4b). Thus, LTD observed under EAAT2 blockade is not dependent on postsynaptic MSN calcium. These results indicate that network effects are involved in LTD expression. They also suggest that VSCCs involved are located on neurons other than the recorded MSN and are activated during EAAT2 blockade, due to glutamate spillover-induced depolarization.

We then investigated the involvement of inhibitory networks in LTD. DHK-induced depolarization would also affect GABAergic interneurons resulting in an increased inhibitory tone^38^. Thus, the observed LTD might arguably arise from an increase in GABA release.

We investigated whether DHK application resulted in an increase in the inhibitory component recorded in MSNs. When MSNs were held at −50 mV, a membrane potential for measuring mainly inhibitory transmission, we observed an outward current of 21±4 pA (*n*=14) (Fig. 4c). In the presence of DHK, this outward current increased by 81%, reaching 37±6 pA, and was inhibited by a GABA_A_R blocker, picrotoxin (50 μM), (PSC after picrotoxin: 12±1 pA; one-way repeated-measures ANOVA: *P*<0.002; *post hoc* Bonferroni-corrected pairwise comparisons: control-DHK: *P*<0.01, DHK-picrotoxin: *P*<0.001). We tested the activation of GABAergic circuits under EAAT2 blockade directly, by making recordings on both striatal fast-spiking (FS) GABAergic interneurons and MSNs during EAAT2 blockade with DHK (Fig. 4d). In brain slices, both FS cells and MSNs are silent at rest, and DHK application led to marked depolarization in both cell types (FS cells: +29±2 mV, *n*=5; MSNs: +24±1 mV, *n*=6; Fig. 4e). Spontaneous firing activity during DHK application was observed only in FS cells (13±7 Hz, *n*=5) whereas MSNs remained silent (Fig. 4f). Cortical stimulation (of an intensity similar to that used for STDP pairings) evoked action potentials in all recorded FS cells whereas MSNs displayed subthreshold EPSPs (Fig. 4f). Thus, DHK application leads to the recruitment of GABAergic interneurons, resulting in an increase of the inhibitory weight exerted on the recorded MSN. An increase in inhibitory drive may, therefore, promote LTD.

We then bath-applied picrotoxin (50 μM) to investigate the involvement of GABAergic networks in LTD. For pairings at −70<Δ*t*_STDP_<+70 ms under EAAT2 blockade, picrotoxin application prevented LTD, instead promoting LTP (202±20%, *P*=0.0075, *n*=6; 6/6 cells displayed LTP; Fig. 4g). These findings suggest that LTD was dependent on GABA_A_R activation. Thus, an increase in inhibitory transmission, probably due to the recruitment of GABAergic interneurons under DHK treatment, is responsible for LTD. Surprisingly, the prevention of this GABAergic inhibition by picrotoxin did not result in the expected lack of plasticity. Instead, it promoted LTP. We analysed the involvement of GABAergic circuits in LTD expression further, by inhibiting GABAergic transmission during transient DHK application. Co-application of gabazine (10 μM; with effects readily reversible by washout) and DHK prevented the expression of plasticity (94±3%, *P*=0.0974, *n*=5; 1/5 cells displayed LTD; Fig. 4h). Thus, GABAergic transmission during STDP pairings is determinant for LTD induction under transient EAAT2 blockade.

The LTD observed under transient EAAT2 blockade, for pairings at −70<Δ*t*_STDP_<+70 ms, is, thus, dependent on the activation of VSCCs, probably located on striatal GABAergic interneurons. The blockade of GABAergic transmission revealed potent LTP, similar to that observed for uncorrelated pairings (−500<Δ*t*_STDP_<−70 ms and +70<Δ*t*_STDP_<+500 ms). Thus, an impairment of EAAT2 function leads to LTP over the entire range of Δ*t*_STDP_, with the exception of a narrow time window (−70<Δ*t*_STDP_<+70 ms), during which GABAergic microcircuits take over LTP and impose LTD.

### Extrasynaptic GluN2B-NMDARs mediate LTP

**Figure 5:**
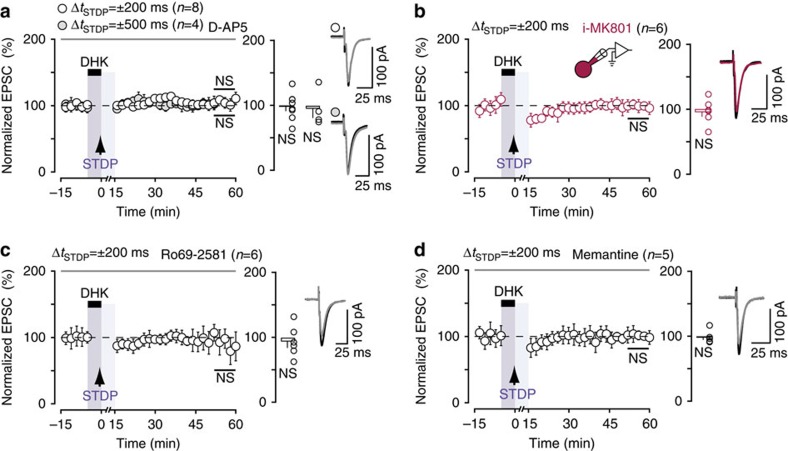
Extrasynaptic GluN2B-NMDARs mediate LTP under transient EAAT2 blockade. (**a**) The LTP induced under EAAT2 blockade for Δ*t*_STDP_=±200 ms and Δ*t*_STDP_=±500 ms was mediated by NMDARs, because it was prevented by D-AP5 (50 μM) application. (**b**) The LTP induced under transient EAAT2 blockade for Δ*t*_STDP_=±200 ms was prevented by blocking postsynaptic NMDARs with i-MK801 (1 mM) applied intracellularly in the recorded MSN. (**c**) The inhibition of GluN2B-containing NMDARs with Ro25–6981 (10 μM) prevented the induction of LTP. (**d**) The inhibition of extrasynaptic NMDARs with memantine (10 μM) prevented LTP under transient EAAT2 blockade. Extrasynaptic GluN2B-containing NMDARs located on the postsynaptic MSN are thus required for the induction of LTP under transient EAAT2 blockade. Insets correspond to the mean of 60 EPSCs during baseline and at 1 h after STDP pairings. Error bars represent s.d. (except in bar graphs: s.e.m.). NS: not significant by one sample *t*-test.

We then investigated the mechanism underlying the LTP observed under transient EAAT2 blockade, for pairings at −500<Δ*t*_STDP_<−70 ms and +70<Δ*t*_STDP_<+500 ms. For both Δ*t*_STDP_=±200 ms and Δ*t*_STDP_=±500 ms, LTP was mediated by NMDAR, as it was prevented by D-AP5 (50 μM; 98±7%, *P*=0.8330, *n*=8; 1/8 cells displayed LTP and 95±14%, *P*=0.7306, *n*=4; 1/4 cells displayed LTP, respectively; Fig. 5a). Glutamate spillover induced by EAAT2 blockade has been reported to mediate crosstalk between neighboring neurons via NMDARs^15^,^40^. We therefore investigated whether the observed LTP was dependent on the recruitment of NMDARs expressed on neighboring cells or solely on the NMDARs located on the postsynaptic MSN subjected to pairings. We used MK801, a use-dependent blocker of NMDARs, which we delivered intracellularly to the postsynaptic MSN used for recording via the patch-clamp pipette (i-MK801; 1 mM). i-MK801 prevented LTP (97±8%, *P*=0.6777, *n*=6; 1/6 cells displayed LTP; Fig. 5b). The NMDARs required for LTP were, therefore, located on the postsynaptic recorded MSN, and not on neighboring cells. We then aimed at identifying further the NMDARs involved in the LTP observed under transient EAAT2 blockade. Glutamate spillover activates high-affinity extrasynaptic NMDARs^14^,^15^,^39^,^40^, which are enriched in the GluN2B subunit^41^. We thus explored the involvement of GluN2B-containing NMDARs in LTP with Ro25–6981, a selective non-competitive antagonist of the GluN2B subunit. Ro25–6981 treatment (10 μM) prevented long-term plasticity (93±10%, *P*=0.5320, *n*=6; 1/6 cells displayed LTP; Fig. 5c), demonstrating the involvement of GluN2B-containing NMDARs in LTP expression under EAAT2 blockade.

The GluN2B subunit is predominantly expressed at extrasynaptic NMDARs but it has also been identified in synaptic NMDARs^41^. We applied memantine (10 μM), a low-affinity uncompetitive NMDAR antagonist that acts as an open-channel blocker with a fast off-rate (see Methods section). Memantine preferentially blocks extrasynaptic NMDARs, without affecting synaptic transmission. Indeed, memantine blocks with a greater extend extrasynaptic NMDARs that are activated due to a low but prolonged elevation of glutamate concentration. By contrast, memantine is relatively inefficient to block NMDARs in the presence of higher synaptic concentrations of glutamate over periods of a few milliseconds, and thus does not interfere with synaptic activity^42^. For STDP during EAAT2 blockade, memantine treatment prevented LTP, as no significant plasticity was observed (99±5%, *P*=0.8302, *n*=5; 1/5 cells displayed LTP; Fig. 5d). Extrasynaptic GluN2B-containing NMDARs located on the postsynaptic recorded striatal MSN are thus required for LTP induction under EAAT2 blockade.

We previously showed that corticostriatal t-LTP is dependent on postsynaptic NMDARs^31^ and, more precisely, that the balance between GluN2A- and GluN2B-containing NMDARs shapes Δ*t*_STDP_^43^. We further investigated whether extrasynaptic NMDARs were required for t-LTP expression in control conditions, as observed for as for LTP observed under EAAT2 blockade. For this purpose, we performed STDP experiments with post–pre pairings at −30<Δ*t*_STDP_<0 ms (similar to the experiments in Fig. 1c,e), in presence of memantine (10 μM); LTP was still observed (222±44%, *P*=0.0271, *n*=8; 7/8 cells displayed LTP; Supplementary Fig. 3). Thus, in control conditions, extrasynaptic NMDARs are not required for t-LTP expression. This finding is consistent with the observation that, compared with t-LTP in control conditions, the LTP induced for uncorrelated pairings under transient EAAT2 blockade involves distinct signalling pathways.

### LTP under transient EAAT2 blockade is not time or orderdependent

**Figure 6:**
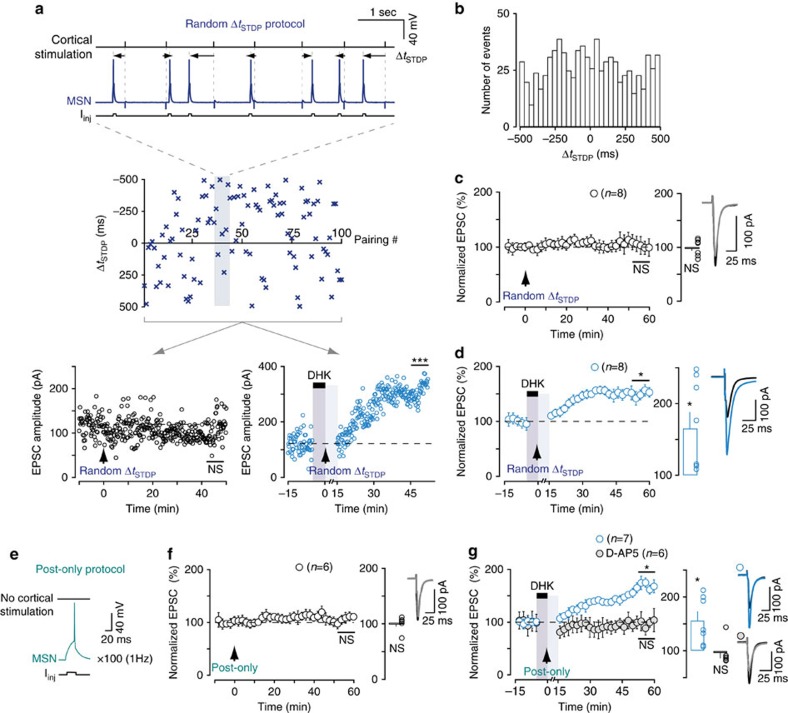
LTP under transient EAAT2 blockade is not dependent on paired activity. (**a**) Example of one random Δ*t*_STDP_ pairing in control conditions and of one such pairing under transient EAAT2 blockade. Top, scatter plot of a single random Δ*t*_STDP_ pattern (comprising 100 consecutive random Δ*t*_STDP_ pairings between −500 and +500 ms) together with the CC traces of 7 successive random pairings. Bottom, examples of experiments performed in two separate MSNs, showing that the same random Δ*t*_STDP_ pattern failed to induce plasticity in control conditions, whereas LTP was observed under transient EAAT2 blockade. (**b**) Histogram of the Δ*t*_STDP_ from the *n*=8 random protocols, showing a uniform distribution. (**b**–**d**) Eight random Δ*t*_STDP_ patterns were generated and each was applied to two MSNs, one in control conditions (**c**) and the other under EAAT2 blockade (**d**). In summary, random Δ*t*_STDP_ patterns failed to induce plasticity in control cells, but resulted in LTP under transient EAAT2 blockade. Thus, under transient EAAT2 blockade, plasticity is not dependent on the timing and order of the paired activity. (**e**) Experimental design depicting a cell conditioning protocol consisting of a postsynaptic spike without paired presynaptic stimulation, repeated 100 times at 1 Hz; (**f**) This protocol did not induce plasticity in control conditions. (**g**) Postsynaptic suprathreshold activity is sufficient to induce potent LTP under transient EAAT2 blockade. This LTP was mediated by NMDARSs, as it was prevented by D-AP5 (50 μM). Insets correspond to the mean of 60 EPSCs during baseline and at 1 h after STDP pairings. Error bars represent the s.d. (except in bar graphs: s.e.m.). *P<0.05; ***P<0.001; NS: not significant by unpaired *t*-test, two-tailed (**a**) or one sample *t*-test (**c**,**d**,**f**,**g**).

Under transient EAAT2 blockade, plasticity was observed even for highly uncorrelated pairings (up to Δ*t*_STDP_=±500 ms; Fig. 2g). This suggests that the induction of plasticity is not dependent on the timing or order of pre- and postsynaptic activity. Timing, order and paired activity are the cardinal features of STDP^11^. We, therefore, investigated whether the plasticity observed under transient EAAT2 blockade nevertheless followed STDP rules. We designed STDP protocols with each of 100 Δ*t*_STDP_ pairings chosen randomly between −500 and +500 ms from a close-to-uniform distribution (see Methods section; Fig. 6). Each of the random pairing protocols (*n*=8) was applied both to a MSN recorded in control conditions and to a MSN subjected to transient EAAT2 blockade. An example is shown in Fig. 6a, with two MSNs (one in control conditions and the other under transient EAAT2 blockade) subjected to the same random pairing template. A single random Δ*t*_STDP_ pattern (taken from the eight different randomly generated Δ*t*_STDP_ patterns) did not trigger plasticity in the MSN in control conditions (the mean baseline EPSC amplitude, 119±3 pA, was not significantly different from the 120±5 pA 1 h after pairings), but it did induce LTP in the MSN subjected to transient EAAT2 blockade (the mean baseline EPSC amplitude, 122±4 pA, increased by 52%, to 307±4 pA, 1 h after pairings). The histogram of the Δ*t*_STDP_ random pairings (*n*=8) in Fig. 6b illustrates that pairings were randomly distributed in a uniform manner. The application of the eight different randomly generated Δ*t*_STDP_ patterns resulted in no significant plasticity in control conditions (99±5%, *P*=0.8429, *n*=8; 2/8 cells displayed LTP; Fig. 6c), whereas these patterns induced LTP under transient EAAT2 blockade (165±22%, *P*=0.0226, *n*=8; 7/8 cells displayed LTP; Fig. 6d). Thus, plasticity under transient EAAT2 blockade does not depend on the timing or order of the paired activity on either side of the synapse and does not, therefore, meet the criteria for STDP.

### LTP under transient EAAT2 blockade does not require paired activity

The timing and order of pairings are crucial for STDP, but were not critical for the expression of plasticity under EAAT2 blockade. We investigated whether paired activity was required to induce plasticity under EAAT2 blockade, by determining whether unpaired activity consisting in postsynaptic spiking (a single postsynaptic action potential repeated 100 times at 1 Hz) without presynaptic stimulation could trigger long-term plasticity (Fig. 6e). In control conditions, this unpaired activity did not induce plasticity (101±5%, *P*=0.9074, *n*=6; 1/6 cells displayed LTP; Fig. 6f). By contrast, under transient EAAT2 blockade, this unpaired activity was sufficient to trigger LTP (156±17%, *P*=0.0152, *n*=7; 6/7 cells displayed LTP; Fig. 6g). This LTP was prevented by D-AP5 (50 μM) and was therefore NMDAR-mediated (96±10%, *P*=0.6693, *n*=6; 1/6 cells displayed LTP; Fig. 6g).

Finally, we investigated whether postsynaptic suprathreshold activity was required to induce plasticity under transient EAAT2 blockade. To do so, we induced subthreshold depolarization (repeated 100 times at 1 Hz without cortical stimulation) in the recorded MSN (Supplementary Fig. 4a). This subthreshold unpaired postsynaptic stimulation was not sufficient to trigger significant plasticity when the average of all experiments performed in these conditions was considered: 118±10% (*P*=0.1213, *n*=6; Supplementary Fig. 4b). However, four of the six recorded MSNs displayed significant LTP (see scatter plot in Supplementary Fig. 4b). The postsynaptic spike therefore seems to be required for the induction of potent NMDAR-mediated LTP under transient EAAT2 blockade.

Correct functioning of EAAT2 is, therefore, required for STDP expression. A cardinal feature for STDP is that it relies on the precise time-correlation between the activities on either side of the synapse. Plasticity under transient EAAT2 blockade therefore does not meet the criteria for STDP.

### EAAT2 overexpression prevents striatal STDP

**Figure 7:**
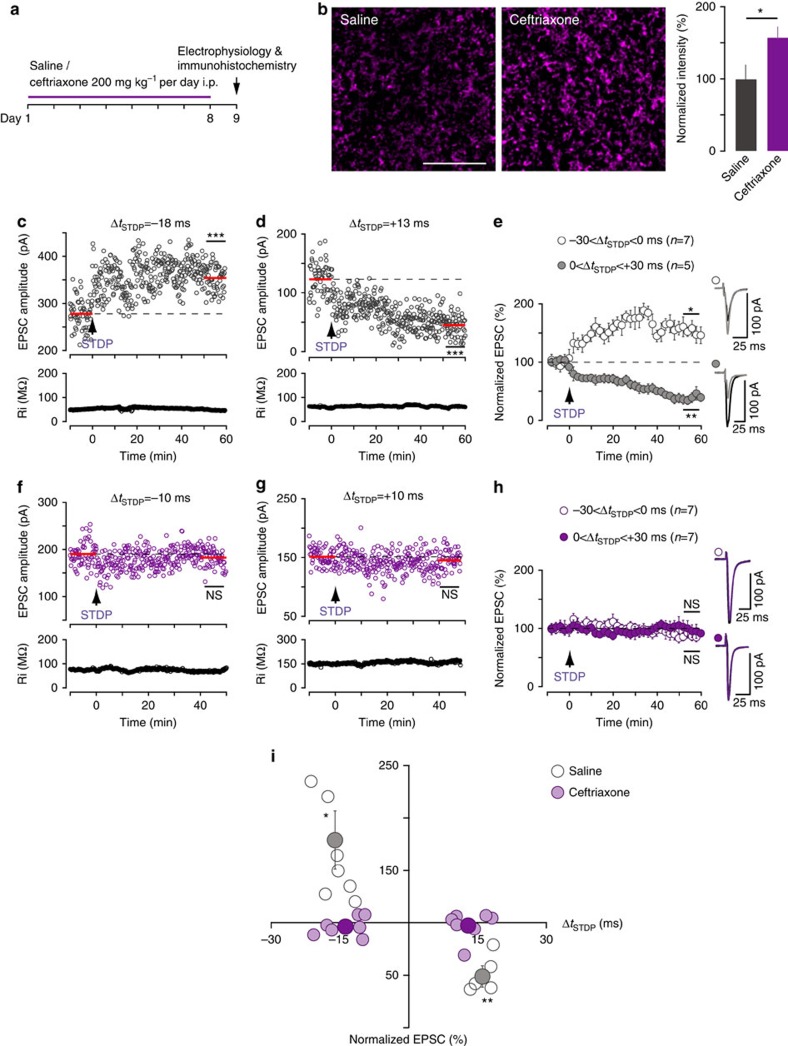
EAAT2 overexpression by ceftriaxone treatment impairs STDP. (**a**) Experimental design: ceftriaxone (or saline) was daily injected for 8 days; electrophysiology and immunohistochemistry experiments were performed 24 h after the last injection. (**b**) Immunohistochemistry revealed an increase of EAAT2-positive puncta in striatal slices from ceftriaxone-injected rats than in slices from saline-injected rats. Scale bar: 10 μm. (**c**) Example of LTP induced by 100 post–pre pairings recorded in a saline-injected rat (Δ*t*_STDP_=−18 ms). Top, EPSC strength before and after pairings. Bottom, time course of Ri (baseline: 50±0.2 MΩ; 50–60 min after pairings: 48±0.2 MΩ; change of −5%). (**d**) Example of LTD induced by 100 pre–post pairings recorded in a saline-injected rat (Δ*t*_STDP_=+13 ms; Ri, baseline: 60±0.3 MΩ; 50–60 min after pairings: 61±0.4 MΩ; change of 0.4%). (**e**) Averaged time-course of experiments performed in saline-injected rats, showing bidirectional STDP: LTP was induced for post–pre (−30<Δ*t*_STDP_<0 ms) and LTD for pre–post (0<Δ*t*_STDP_<+30 ms) pairings. (**f**) Example of the lack of plasticity observed with 100 post–pre pairings (Δ*t*_STDP_=−10 ms) recorded from a ceftriaxone-treated rat. Top, EPSC strength was not significantly different before and after pairings. Bottom, time course of Ri (baseline: 75±0.3 MΩ; 40–50 min after pairings: 69±0.5 MΩ; change of −8%). (**g**) Example of the absence of plasticity observed with 100 pre–post pairings (Δ*t*_STDP_=+10 ms) from a ceftriaxone-treated rat. EPSC strength did not differ significantly before and after pairings (Ri, baseline: 149±0.6 MΩ; 40–50 min after pairings 163±10 MΩ; change of 10%). (**h**) Averaged time course of experiments performed on ceftriaxone-treated rats, showing an absence of STDP for both post–pre and pre–post pairings. (**i**) Time window for long-term synaptic strength for post–pre and pre–post pairings (−30<Δ*t*_STDP_<+30 ms) in saline- and ceftriaxone-treated rats. Synaptic strength was assessed 45–60 min after pairings (empty and pink circles: individual neurons; gray or purple circles: average). Bidirectional plasticity was induced in saline-injected rats, whereas no plasticity was observed in ceftriaxone-treated rats. Insets correspond to the average of 60 EPSCs during baseline and at 1 h after STDP pairings. Error bars represent the s.d. (except in panel **b**,**i**: s.e.m.). *P<0.05; **P<0.01; ***P<0.001; NS: not significant by unpaired *t*-test, two-tailed (**b**–**d**,**f**,**g**) or one sample *t*-test (**e**,**h**,**i**).

To estimate to what extent EAAT2 controls STDP expression, we next questioned if an overexpression of EAAT2 would have an impact on STDP. We used ceftriaxone, a beta-lactam antibiotic that increases EAAT2 levels and activity^44^. Indeed, immunohistochemistry showed that eight days of daily i.p. ceftriaxone (200 mg kg^−1^) injections in rats (Fig. 7a) significantly increased (*P*=0.0420) EAAT2 levels in the striatum (Fig. 7b). The control group consisted of rats receiving a daily injection of an equal volume of saline for 8 days. We observed no significant difference between saline- and ceftriaxone-injected rats for passive and active membrane properties of MSNs (RMP, Ri, rheobase, intensity-frequency relationship), transmission and short-term plasticity (Supplementary Fig. 5a–i) or NMDAR-mediated EPSCs (Supplementary Fig. 5j–l). We verified that similar STDP was observed in saline-injected and control rats. The examples in Fig. 7c,d show that post–pre pairings at Δ*t*_STDP_=−18 ms induced LTP (the mean baseline EPSC amplitude was 278±4 pA before pairings and had increased by 27%, to 354±3 pA, 1 h after pairings; Fig. 7c) whereas pre–post pairings at Δ*t*_STDP_=+13 ms induced LTD (the mean baseline EPSC amplitude was 123±4 pA before pairings and had decreased by 63%, to 45±2 pA, 1 h after pairings; Fig. 7d). In summary, saline-injected rats displayed bidirectional STDP similar to that observed in control rats: post–pre pairings induced LTP (179±28%, *P*=0.0295, *n*=7; 7/7 cells displayed LTP) and pre–post pairings triggered LTD (51±8%, *P*=0.0036, *n*=5; 5/5 cells displayed LTD; Fig. 7e,i). In ceftriaxone-treated rats, canonical pairings were unable to induce STDP. Indeed, as exemplified in Fig. 7f, post–pre pairings at Δ*t*_STDP_=−10 ms failed to induce plasticity: no significant difference was observed before and after pairings (190±3 pA and 182±3 pA, respectively). Similarly, an absence of plasticity was observed for pre–post pairings at Δ*t*_STDP_=+10 ms because there was no significant difference before and after pairings (151±2 pA and 148±3 pA, respectively; Fig. 7g). In summary, MSNs recorded from ceftriaxone-treated rats displayed no STDP as both post–pre (−30<Δ*t*_STDP_<0 ms) and pre–post (0<Δ*t*_STDP_<+30 ms) pairings failed to induce significant plasticity (96±3%, *P*=0.3286, *n*=7, 0/7 cells displayed LTP and 97±5%, *P*=0.6279, *n*=7, 1/7 cells displayed LTD, respectively; Fig. 7h,i). In conclusion, EAAT2 overexpression impaired the detection of correlated activity and precluded the occurrence of a bidirectional STDP (Fig. 7i).

## Discussion

**Figure 8:**
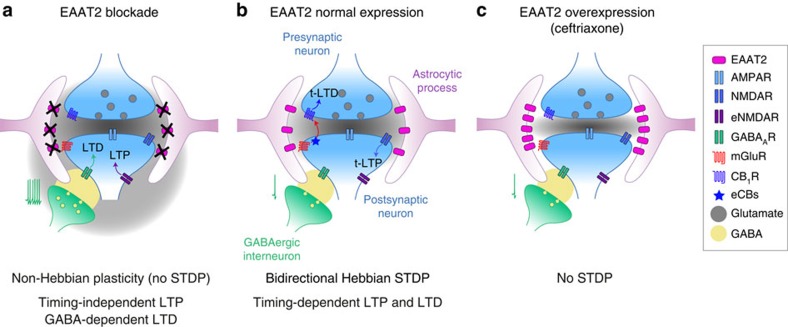
Impact of astrocytes, via EAAT2, on Hebbian plasticity. Schematic representation of the role of astrocytes, via EAAT2, on Hebbian plasticity in the striatum. (**a**) Transient EAAT2 blockade prevents the expression of STDP, instead favoring non-Hebbian plasticity (timing-independent LTP). This LTP is mediated by extrasynaptic NMDAR and LTD is dependent on the activation of striatal GABAergic microcircuits. In these conditions, unpaired activity is sufficient to induce LTP. (**b**) The physiological expression and activity of EAAT2 allows the emergence of Hebbian plasticity (bidirectional STDP). Depending on the order of pre- and postsynaptic activity, NMDAR-mediated t-LTP or endocannabinoid-mediated t-LTD is induced. (**c**) EAAT2 overexpression by limiting glutamate spillover prevents STDP expression. Thus, the efficiency of glutamate uptake, most through astrocytic EAAT2, gates the expression of Hebbian synaptic plasticity in the striatum.

Identifying the conditions required for the expression of Hebbian plasticity, such as STDP, is essential for a better understanding of the mechanisms underlying learning and memory. Our findings demonstrate that astrocytes play a key role in the establishment of STDP, through EAAT2-mediated glutamate uptake. Indeed, EAAT2 allows translating precise pre- and postsynaptic activity into a salient time-coded message. This is a key requirement for STDP, the main characteristic of which is a high degree of sensitivity to timing^19^,^20^, a feature that was erased by the transient blockade of EAAT2. Under this blockade, STDP was replaced by a non-Hebbian form of plasticity that was not dependent on the timing or order of the activities on either side of the synapse and was even observed in cases of unpaired activity. By contrast, EAAT2 overexpression impaired the detection of correlated pre- and postsynaptic activity by MSNs, resulting in an absence of plasticity. Our results show that astrocytes gate the conversion from non-Hebbian to Hebbian plasticity via EAAT2, leading to the emergence of STDP (Fig. 8).

Astrocytes actively control various synaptic functions and, therefore, play a key role in the modulation of neuronal activity^11^,^12^,^45^,^46^. Control of neuronal computation by astrocytes is via the release and uptake of transmitters, such as glutamate. Glutamate release by astrocytes plays an important role in STDP at L4-L2/3 neocortical synapses, by controlling t-LTD through the activation of astrocytic CB_1_R^47^. By contrast, the involvement of astrocytic glutamate uptake in a time-coding paradigm, such as STDP, has never been investigated. Previous reports indicate that rate-coded plasticity, induced by low- or high-frequency stimulation (LFS and HFS) or theta-burst stimulation (TBS), is sensitive to changes in astrocytic glutamate uptake^48^,^49^,^50^,^51^,^52^,^53^. In addition, neuronal EAAT3 regulates the balance between TBS-LTP and LFS-LTD^54^ and cerebellar LTD is dependent on the patterned expression of neuronal EAAT4 on Purkinje cells^55^. This study is, to our knowledge, the first to assess the involvement of astrocytic glutamate uptake in the expression of time-coded plasticity. STDP relies on the precise timing and order of inputs on either side of the synapse and thus constitutes a time-coding paradigm for plasticity induction^19^,^20^ by contrast to rate-coding plasticity protocols. The detection of a temporal coincidence between pre- and postsynaptic activities is crucial for STDP expression. Astrocytic glutamate uptake is involved in setting the timing of synaptic inputs. We therefore explored the role of EAAT2 in STDP, by transiently inhibiting (with DHK or WAY-213,613) EAAT2 during STDP pairings. This allows an on-off manipulation compatible with STDP study, whereas genetic approaches (knockout) and long-lasting drug applications have potential long-term effects. DHK and WAY-213,613 have several advantages for studies of this type. In addition to their specificity for EAAT2 and their efficient washout, they are also non-transportable inhibitors of EAAT2, and this property prevents artificial increases in extracellular glutamate concentration due to hetero-exchange^33^,^34^. We next overexpressed EAAT2 with ceftriaxone, which has been reported to increase EAAT2 expression and activity^44^.

Astrocytic pools of EAAT2 are responsible for 90% of the glutamate uptake^8^. EAAT2 is also found on neurons but at much lower level (∼10% of astrocytic EAAT2). The physiological role of neuronal EAAT2 remains uncertain based on their very low level of expression but also on their distribution in most of the axon-terminal membranes and not being concentrated in the synapses^9^,^56^. Specific deletion of EAAT2 in astrocytes induces dramatic effects, such as excess mortality, lower body weight and spontaneous seizures, whereas no detectable neurological abnormalities are observed with neuronal EAAT2 deletion^8^,^9^.

The key feature of STDP is its occurrence within a restricted time window. Uncorrelated events (>30 ms) therefore fail to trigger plasticity. When EAAT2 activity is transiently impaired, an aberrant form of plasticity occurs during time windows in which plasticity is not normally observed. Uncorrelated events can induce this aberrant plasticity and are considered as pertinent events for an engram. Unlike STDP, the non-Hebbian LTP induced under transient EAAT2 blockade did not depend on the timing or order of pre- and postsynaptic activity. t-LTP has been reported to be mainly dependent on NMDARs^19^, which operate as molecular coincidence detectors^4^. By contrast, non-Hebbian LTP under EAAT2 blockade is dependent on postsynaptic GluN2B-containing NMDARs located extrasynaptically, and these receptors do not act as molecular coincident detectors. Supporting this, we found that even unpaired activity (consisting of a single postsynaptic action potential repeated 100 times at 1 Hz) induced non-Hebbian LTP under EAAT2 blockade (Fig. 6g). Molecular coincidence detectors, such as NMDARs, require concomitant signals to be activated, as in STDP, in which the postsynaptic back-propagating action potential is paired with presynaptic activity^19^,^20^. In the presence of transient EAAT2 blockade, this feature is lost, because a single signal, the postsynaptic back-propagating action potential removing Mg^2+^ blockade, becomes sufficient to trigger LTP, due to the high ambient glutamate levels present when EAAT2 is blocked.

GABAergic microcircuits are involved in plasticity occurring at specific time window (−70<Δ*t*_STDP_<+70 ms) resulting in LTD (by contrast to the non-timing-dependent LTP). In the presence of DHK, GABAergic inhibition was stronger, due to the recruitment of inhibitory neurons as a result of the increase in glutamate spillover. In the presence of blockers of GABA_A_Rs or VSCCs, pairings for which −70<Δ*t*_STDP_<+70 ms unmasked NMDAR-mediated LTP.

We previously described the control of STDP polarity by GABA^28^. Here, different mechanisms are involved because concomitant transient blockade of GABAergic transmission and EAAT2 led to an absence of plasticity. GABAergic circuits are efficiently recruited by cortical stimulation in the presence of DHK. We hypothesize that the NMDAR-mediated LTP observed at large Δ*t*_STDP_ is somehow shunted at narrow Δ*t*_STDP_ by an additional pool of GABA, due to the recruitment of GABAergic interneurons by cortical stimulation. Indeed, NMDAR-mediated LTP at larger Δ*t*_STDP_ was exclusively dependent on the postsynaptic spiking (Fig. 6g) and did not require presynaptic stimulation. By contrast, when cortical stimulation (and, thus, the recruitment of GABAergic interneurons) was paired with the postsynaptic spike for narrow Δ*t*_STDP_, the increased GABAergic transmission prevented LTP expression. Thus, NMDAR-mediated LTP may be expressed only at large Δ*t*_STDP_, when presynaptic stimulation occurs far from the postsynaptic spike and GABAergic evoked transmission does not interfere with LTP expression. As a result, the blocking of GABA_A_R transmission revealed LTP. This LTP was similar to the non-timing-dependent LTP (NMDAR-mediated) induced for large Δ*t*_STDP_. Interestingly, pre–post t-LTD and post–pre t-LTP observed in control conditions are both dependent on VSCC activity^31^, but their induction itself is not dependent on GABAergic transmission^28^. Thus, the t-LTD and t-LTP evoked in control conditions involve signalling mechanisms distinct from those involved in the plasticity observed under EAAT2 blockade.

EAAT2 overexpression by ceftriaxone prevented both t-LTP and t-LTD. We verified that ceftriaxone did not alter the passive and active electrophysiological properties of MSNs, as well as corticostriatal transmission and probability of glutamate release. Ceftriaxone can also mediate the upregulation of system x_c_- (cystine/glutamate antiporter system)^57^, which, together with EAAT2, is involved in the maintenance of glutamate homeostasis. However, the net effect of up or downregulation and the precise balance between these two systems (glutamate uptake and export) remains to be determined. We have previously shown that the bidirectional corticostriatal STDP relies on two distinct signalling pathways^31^,^43^. Indeed, t-LTP is NMDAR-dependent, whereas t-LTD is mGluR-mediated. Both receptor subtypes can be localized outside the synaptic cleft^37^,^41^ and thus compete with EAAT2 for the extracellular glutamate. Overexpression of EAAT2 by ceftriaxone is expected to enhance glutamate uptake and reduce spillover. This would reasonably result in a profound alteration of corticostriatal STDP expression. We hypothesize that enhanced glutamate clearance by EAAT2 upregulation may prevent the activation of postsynaptic NMDARs or type-ImGluRs, leading to the lack of t-LTP or t-LTD, respectively. In line with that, increases in glutamate transporter expression have been shown to alter frequency-based plasticity dependent on glutamate spillover, such as mGluR-mediated LFS-LTD and HFS-LTP in the hippocampus^53^. However, ceftriaxone has been mainly used in neurodegeneration and addiction models where extracellular glutamate levels are greatly enhanced^10^,^12^. Importantly, ceftriaxone abolishes the increase in glutamate spillover (assessed by NMDAR-EPSCs) in heroin-treated animals but not in control yoked saline animals^58^. In agreement with this^58^, we did not detect significant difference between NMDAR-EPSCs decay in saline and ceftriaxone-treated rats (Supplementary Fig. 5j–l). One possible explanation is that monitoring NMDAR-EPSCs does not allow differentiating between synaptic and extrasynaptic NMDARs. Therefore, the fraction of ambient versus synaptic glutamate detected by extrasynaptic NMDARs is difficult to assess. We hypothesize that under EAAT2 blockade, a critical number of peri- and/or extrasynaptic NMDARs are recruited leading to non-Hebbian plasticity. On the contrary, EAAT2 overexpression would reduce the pool of activated peri- and/or extrasynaptic NMDARs and consequently prevents STDP expression.

A few studies have reported effects of changes in EAAT2 expression on behaviour^46^. The pharmacological blockade of EAAT2 with DHK impairs spatial memory and induces depression and anhedonia and ceftriaxone has been reported to display antidepressant effects^46^. EAAT2 downregulation in striatum is also found in a rat model of depression^59^. EAAT2 KO mice exhibit seizures and premature death^6^,^9^. An inducible astrocytic EAAT2 knockout was recently shown to be associated with pathological repetitive behaviours and an increase in corticostriatal excitatory transmission^25^. Moreover, this phenotype was reversed by memantine treatment, confirming that excessive glutamate spillover due to EAAT2 dysfunction, deregulating the corticostriatal pathway, was responsible for the observed repetitive behaviours. These findings are consistent with our results showing that memantine prevents aberrant LTP in conditions of EAAT2 blockade. Conversely, EAAT2 overexpression has been reported to impair hippocampal learning^60^. This observation is consistent with our results showing a lack of plasticity with ceftriaxone treatment.

EAAT2 dysfunction, associated with higher ambient glutamate levels, has been observed in neurodegenerative and psychiatric diseases including Huntington's, Parkinson's, Alzheimer's and schizophrenia in which cognitive functions are impaired^7^,^10^,^11^. Chronic exposure to drugs of abuse has also been shown to induce a downregulation of EAAT2 in the nucleus accumbens^12^. EAAT2 therefore appears to be a major target for the treatment of neurological diseases and addiction (by ceftriaxone), not only to combat glutamatergic neurotoxicity but also to prevent aberrant plasticity, which could be linked to cognitive deficits^10^,^11^,^12^. Thus, our results, showing the tight control of STDP by EAAT2, are of importance for linking the expression of timing-dependent plasticity with different physiological or pathological states.

Astrocyte function is not restricted to structural and metabolic support or homeostatic and protective functions. Through glutamate uptake, astrocytes are also involved in higher brain functions, such as learning and memory^11^,^45^,^46^. We demonstrate here that EAAT2 operates over a highly controlled range to allow the emergence of bidirectional STDP. If STDP is dependent on the efficiency of glutamate uptake, then we would expect STDP expression to be controlled by the precise location and density of transporter expression, and glial synaptic coverage, which may differ considerably between brain structures and can undergo experience-dependent remodelling^61^ (Fig. 8). This work thus identifies astrocytes as key players in the establishment of synaptic Hebbian learning rule, such as STDP.

## Methods

### Animals

All experiments were performed in accordance with the guidelines of the local animal welfare committee (Center for Interdisciplinary Research in Biology Ethics Committee) and the EU (directive 2010/63/EU). Every precaution was taken to minimize stress and the number of animals used in each series of experiments. OFA rats P18–42 (Charles River, L'Arbresle, France) were used for brain slice electrophysiology. Animals were housed in standard 12-h light/dark cycles and food and water were available *ad libitum*.

### Brain slice preparation

Horizontal brain slices containing the somatosensory cortical area and the corresponding corticostriatal projection field were prepared as previously described^27^,^28^,^31^. Corticostriatal connections (between somatosensory cortex layer 5 and the dorsolateral striatum) are preserved in the horizontal plane. Horizontal brain slices (330 μm-thick) were prepared from rats with a vibrating blade microtome (VT1200S, Leica Micosystems, Nussloch, Germany). Brains were sliced in an ice-cold cutting solution (125 mM NaCl, 2.5 mM KCl, 25 mM glucose 25 mM NaHCO_3_, 1.25 mM NaH_2_PO_4_, 2 mM CaCl_2_, 1 mM MgCl_2_, 1 mM pyruvic acid) through which 95% O_2_/5% CO_2_ was bubbled. The slices were transferred to the same solution at 34 °C for 1 h and then to room temperature.

### Electrophysiology recordings

Patch-clamp recordings were performed as previously described^27^,^28^,^31^. Briefly, for whole-cell recordings, borosilicate glass pipettes of 6–8 MΩ resistance were filled with (in mM): 105 K-gluconate, 30 KCl, 10 HEPES, 10 phosphocreatine, 4 Mg-ATP, 0.3 Na-GTP, 0.3 EGTA (adjusted to pH 7.35 with KOH). The composition of the extracellular solution was (mM): 125 NaCl, 2.5 KCl, 25 glucose, 25 NaHCO_3_, 1.25 NaH_2_PO_4_, 2 CaCl_2_, 1 MgCl_2_, 10 μM pyruvic acid bubbled with 95% O_2_ and 5% CO_2_. Signals were amplified using with EPC9-2 and EPC10-4 amplifiers (HEKA Elektronik, Lambrecht, Germany). All recordings were performed at 34 °C, using a temperature control system (Bath-controller V, Luigs and Neumann, Ratingen, Germany) and slices were continuously superfused with extracellular solution, at a rate of 2 ml min^−1^. Slices were visualized under an Olympus BX51WI microscope (Olympus, Rungis, France), with a 4 × /0.13 objective for the placement of the stimulating electrode and a 40 × /0.80 water-immersion objective for the localization of cells for whole-cell recordings. Current–clamp recordings were filtered at 2.5 kHz and sampled at 5 kHz and voltage–clamp recordings were filtered at 5 kHz and sampled at 10 kHz, with the Patchmaster v2 × 32 program (HEKA Elektronik).

To compare the decay time of NMDAR-mediated EPSCs in saline- and ceftriaxone-treated animals, whole-cell recordings in voltage–clamp mode were performed at +40 mV clamping voltage. Borosilicate glass pipettes were filled with (in mM): 124 cesium, 10 NaCl, 1 MgCl_2_, 10 HEPES, 1 EGTA, 1 QX, 2 Mg-ATP, 0.3 Na-GTP (adjusted to pH 7.35 with CsOH). The stimulation protocol used for triggering NMDAR-EPSCs consisted in eight presynaptic stimulation pulses elicited at 100 Hz.

### Spike timing-dependent plasticity protocols and random Δ*t*_STDP_ patterns

Electrical stimulations were performed with a concentric bipolar electrode (Phymep, Paris, France and CBBSE75 FHC, Bowdoin, ME, USA) placed in layer 5 of the somatosensory cortex^27^. Electrical stimulations were monophasic, at constant current (ISO-Flex stimulator, AMPI, Jerusalem, Israel). Currents were adjusted to evoke 100–400 pA EPSCs. Repetitive control stimuli were applied at 0.1 Hz. STDP protocols consisted of pairings of pre- and postsynaptic stimulations (at 1 Hz) separated by a specific time interval (Δ*t*_STDP_). Presynaptic stimulations corresponded to cortical stimulations and the postsynaptic stimulation of an action potential evoked by a depolarizing current step (30 ms duration) in MSNs. Δ*t*_STDP_<0 ms for post–pre pairings, and Δ*t*_STDP_>0 ms for pre–post pairings. Δ*t*_STDP_=±500 ms corresponds to post–pre and pre–post pairings performed around Δ*t*_STDP_=−500 ms and Δ*t*_STDP_=+500 ms. Note that for Δ*t*_STDP_=−500 ms and Δ*t*_STDP_=+500 ms, the order (post–pre versus pre–post) was determined by the first pairing of the STDP protocol only, because, for the remaining pairings, the pre- and postsynaptic stimulations were separated by 500 ms and could therefore be considered as either post–pre or pre–post pairings at 1 Hz. We therefore pooled the data for Δ*t*_STDP_=−500 ms and Δ*t*_STDP_=+500 ms (Δ*t*_STDP_=±500 ms), which are presented as a single average on the figures. Recordings on neurons were made over a period of 10 min at baseline, and for at least 50 min after the SDTP protocols; long-term changes in synaptic efficacy were measured for the last 10min. We individually measured and averaged 60 successive EPSCs, comparing the last 10 min of the recording with the 10-minute baseline recording. Whole-cell recordings were made in voltage–clamp mode during baseline and for the 60 min of recording after the STDP protocol, and in current–clamp mode during STDP protocol. Experiments were excluded if input resistance (Ri) varied by more than 20%.

For the random Δ*t*_STDP_ patterns, we used the following algorithm (programmed in Igor Pro 6.3 software, WaveMetrics): for each pairing, we first selected a time window with a randomly selected length between 500 and 1,500 ms (with a uniform distribution) and located the presynaptic stimulation time in the middle of this window. The postsynaptic stimulation time was then randomly chosen within this window (with a uniform distribution). The Δ*t*_STDP_ pattern was formed by the concatenation of 100 such windows. This generated both a close-to-uniform distribution of the Δ*t*_STDP_ and a variable interval between two successive presynaptic stimulations.

### Chemicals

All chemicals were purchased from Tocris (Ellisville, MO, USA), except for picrotoxin (Sigma). (2S,3S,4R)-2-Carboxy-4-isopropyl-3-pyrrolidineacetic acid (Dihydrokainic acid, DHK; 300 μM), DL-2-amino-5-phosphono-pentanoic acid (D-AP5; 50 μM), (1S,2S)-2-[2-[[3-(1H-benzimidazol-2yl)propyl]methylamino]ethyl]-6-fluoro-1,2,3,4-tetrahydro-1-(1-methylethyl)-2-naphthalenyl methoxyacetoacetate dihydrochloride (Mibefradil; 20 μM), 6-cyano-7-nitroquinoxaline-2,3-dione (CNQX; 20 μM), (αR,βS)-α-(4-hydroxyphenyl)-β-methyl-4-(phenylmethyl)-1-piperidinepropanol maleate (Ro 25–6981; 10 μM), SR 95531 hydrobromide (gabazine; 10 μM) and 3,5-dimethyl-tricyclo[3.3.1.13,7]decan-1-amine hydrochloride (Memantine; 10 μM) were dissolved directly in the extracellular solution and bath applied. N-(piperidin-1-yl)-5-(4-iodophenyl)-1-(2,4-dichlorophenyl)-4-methyl-1H-pyrazole-3-carboxamide (AM251; 3 μM) and picrotoxin (50 μM) were dissolved in ethanol and added to the external solution, such that the final concentration of ethanol was 0.01–0.1%. N-[4-(2-bromo-4,5-difluorophenoxy)phenyl]-L-asparagine (WAY-213,613; 50 and 100 μM) was dissolved in DMSO and added to the external solution such that the final concentration of DMSO was 0.05 and 0.1%, respectively. (S)-α-Methyl-4-carboxyphenylglycine (MCPG; 500 μM) was dissolved in 1.1 eq. NaOH and added to the external solution. BAPTA (10 mM) and dizocilpine maleate (i-MK801; 1 mM) were dissolved directly in the intracellular solution.

The contrasting activity patterns of synaptic and extrasynaptic NMDARs result in different degrees of memantine blockade^42^. Due to the agonist concentration-dependence of memantine blockade kinetics, slices were pre-incubated with low dose of memantine (10 μM) for at least 1 h before recording, to allow sufficient time for equilibrium to be reached.

### Transient EAAT2 blockade

Transient EAAT2 blockade was achieved with two structurally different molecules: DHK (300 μM)^33^ and WAY-213,613 (50–100 μM)^34^, which are both selective non-substrate inhibitors (non-transportable) of EAAT2. DHK was bath-applied for as short a period as possible, to ensure that its effect on Vm was compatible with the correct analysis of synaptic efficacy changes. Indeed, EAAT2 blockade resulted in a marked depolarization^26^, potentially impairing the estimation of synaptic efficacy changes. A stable baseline was established over a period of 10 min. DHK was bath-applied for 5 min (the dark gray area in the figures). We systematically checked the efficacy of DHK application before applying the STDP protocol. This depolarization (Fig. 2a) was used as an indicator of DHK efficiency. DHK was washed out at the STDP protocol offset. The full DHK washout took 15 min (the light gray area in the figures) and, during this period, a significant and transient decrease in EPSC magnitude (due to the DHK-induced inward shift in *I*_holding_ and AMPAR desensitization^26^) was observed. Accordingly, in all figures, synaptic efficacy changes are illustrated from 15 min after the removal of DHK. Synaptic efficacy changes were evaluated 50–60 min after the start of the DHK washout (at least 30 min after the full recovery of baseline *I*_holding_).

### Electrophysiological data analysis

Off-line analysis was performed with Fitmaster (Heka Elektronik). Spontaneous post-synaptic currents (sPSCs) were identified using a semi-automated amplitude threshold based detection software (Mini Analysis 6.0.7 Program, Synaptosoft, Fort Lee, NJ, USA) and were visually confirmed. Analysis of NMDAR-EPSCs was performed using a custom-build analysis in Python. After removal of the stimulation artifacts, NMDAR-EPSCs decay was normalized and fitted to a bi-exponential curve. The fast and slow decay times of NMDAR-EPSCs (tau1 and tau2, respectively) were then quantified. Statistical analysis was performed with Prism 5.02 software (San Diego, CA, USA). In all cases ‘*n*' refers to an experiment on a single cell from a single slice. In average, 2 cells per animal were obtained. All results are expressed as mean±s.e.m. in the text and in the figures (except as mean±s.d. in the figures for plasticity graphs: normalized EPSC versus time), and statistical significance was assessed in unpaired *t*-tests or in one-sample *t*-tests, as appropriate, using the indicated significance threshold (*P*), or one-way ANOVA with Bonferroni correction, where specified.

### Chronic ceftriaxone treatment

To increase the expression of EAAT2 chronic ceftriaxone treatment of the rats was performed as previously described^53^. Male OFA rats (P30-P42) received a daily intraperitoneal (i.p.) injection of ceftriaxone (Rocefin, Roche; 200 mg kg^−1^ per day dissolved in saline) or an equal volume of saline on eight consecutive days. Corticostriatal brain slices for electrophysiology were obtained from ceftriaxone- or saline-treated rats 24 h after the final injection, and prepared as described above.

### Immunohistochemistry

Rats were treated for eight days with daily i.p. injection of either saline (*n*=4 rats) or ceftriaxone (*n*=4 rats), as described above. Rats were anesthetized with pentobarbital. Brains were postfixed in 4% paraformaldehyde and cut into 30 μm horizontal sections with a vibratome (Microm HM650V, ThermoScientific). Immunostaining was performed by incubating free-floating sections with a guinea pig anti-EAAT2 antibody (1:5000; AB1783, Merck Millipore) for 48 h at 4 °C and then with a secondary Cyanine Cy3-conjugated antibody (1:1,000; Jackson Laboratories) dissolved in PBS 1X for 1 h. Images were acquired with the SP5 confocal system (Leica, Germany).

### Data availability

All relevant data are available from the authors.

## Additional information

**How to cite this article:** Valtcheva, S. & Venance, L. Astrocytes gate Hebbian synaptic plasticity in the striatum. *Nat. Commun.* **7,** 13845 doi: 10.1038/ncomms13845 (2016).

**Publisher's note:** Springer Nature remains neutral with regard to jurisdictional claims in published maps and institutional affiliations.

## Supplementary Material

Supplementary Information Supplementary Figures.
