# Supplementary material for: Astrocytes gate Hebbian synaptic plasticity in the striatum
Source: Nat Commun. 2016 Dec 20;7:13845. doi: 10.1038/ncomms13845 (PMC5187441; doi:10.1038/ncomms13845)
Supplement: Supplementary Information — Supplementary Figures. [file ncomms13845-s1.pdf]

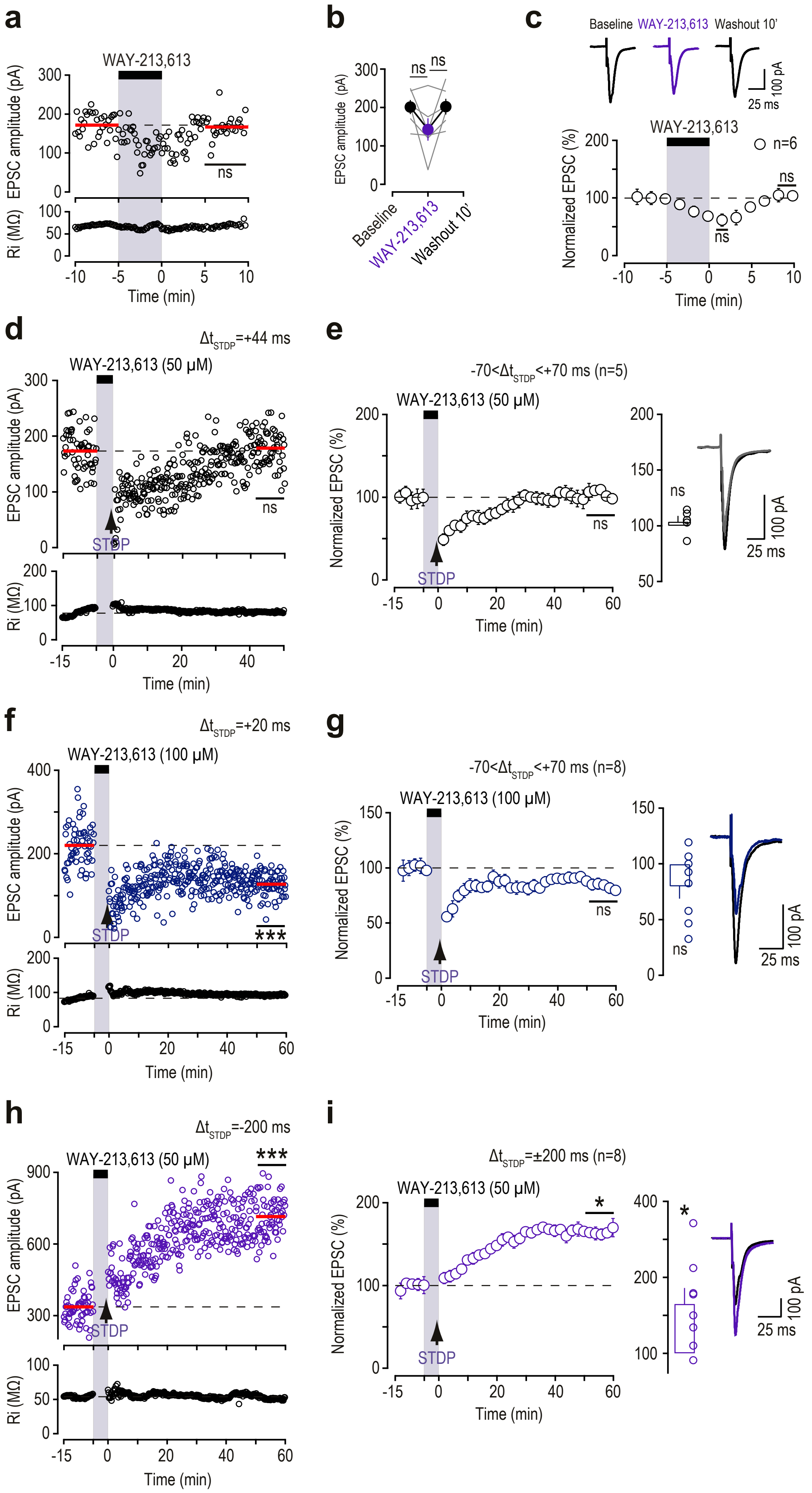
a
WAY-213,613
EPSC amplitude (pA)
Ri (MΩ)
ns
Time (min)
b
EPSC amplitude (pA)
ns
ns
Baseline
WAY-213,613
Washout 10'
c
Baseline
WAY-213,613
Washout 10'
100 pA
25 ms
WAY-213,613
n=6
Normalized EPSC (%)
ns
ns
Time (min)
d
Δt_{STDP}=+44 ms
WAY-213,613 (50 μM)
EPSC amplitude (pA)
ns
STDP
Ri (MΩ)
Time (min)
e
-70<Δt_{STDP}<+70 ms (n=5)
WAY-213,613 (50 μM)
Normalized EPSC (%)
ns
STDP
Time (min)
ns
100 pA
25 ms
f
Δt_{STDP}=+20 ms
WAY-213,613 (100 μM)
EPSC amplitude (pA)
STDP
***
Ri (MΩ)
Time (min)
g
-70<Δt_{STDP}<+70 ms (n=8)
WAY-213,613 (100 μM)
Normalized EPSC (%)
ns
STDP
Time (min)
ns
100 pA
25 ms
h
Δt_{STDP}=-200 ms
WAY-213,613 (50 μM)
***
EPSC amplitude (pA)
STDP
Ri (MΩ)
Time (min)
i
Δt_{STDP}=±200 ms (n=8)
WAY-213,613 (50 μM)
*
Normalized EPSC (%)
STDP
Time (min)
*
100 pA
25 ms

Supplementary Figure 1. The transient inhibition of EAAT2 by WAY-213,613 disrupts STDP

(**a-c**) WAY-213,613 application had no effect on the changes in synaptic efficacy estimated from WAY-213,613 washout (example in a, and averaged time-course of experiments in b and c). The brief application of WAY-213,613 induced a non-significant transient decrease in EPSC amplitude, with no change in Ri. The effect of WAY-213,613 on synaptic transmission was, thus, compatible with the estimation of long-term synaptic efficacy changes. (**d**) Example of the lack of plasticity observed with 100 pre-post pairings (t_{STDP}=+44 ms) during the transient blockade of EAAT2 with WAY-213,613 (50 μM for 5 min, gray area). Top, EPSC strength before and after pairings. Bottom, time course of Ri (baseline, 79±1MΩ; 50-60 min after pairings, 81±0.2MΩ; change of 2%). (**e**) Averaged time-course of experiments with a transient blockade of EAAT2 with WAY-213,613 (50 μM), with the absence of plasticity induction for pairings at -70< t_{STDP}<+70 ms. (**f**) Example of LTD induced by 100 pre-post pairings (t_{STDP}=+20 ms) with a transient blockade of EAAT2 with WAY-213,613 (100 μM). Top, EPSC strength before and after pairings. Bottom, time course of Ri (baseline, 84±0.2MΩ; 50-60 min after pairings, 92±0.2MΩ; change of 11%). (**g**) Averaged time-course of experiments with a transient blockade of EAAT2 with WAY-213,613 (50 μM), with no significant induction of plasticity for pairings at -70< t_{STDP}<+70 ms. However, it should be noted that LTD was more frequent (5/8 cells) when induced with 100 μM WAY-213,613 than when induced with 50 μM WAY-213,613 (1/5 cells). (**h**) Example of LTP induced by 100 post-pre pairings (t_{STDP}=-200 ms) with a transient blockade of EAAT2 with WAY-213,613 (50 μM) (Ri, baseline: 54±0.3MΩ; 50-60 min after pairings: 52±0.3MΩ; change of -4%). (**h**) Example of LTP induced by 100 post-pre pairings (t_{STDP}=-200 ms) with a transient blockade

of EAAT2 with WAY-213,613 (50 μM). Top, EPSC strength before and after pairings. Bottom, time course of Ri (baseline, 54±0.3MΩ; 50-60 min after pairings, 52±0.3MΩ; change of -4%). (**i**) Averaged time-course of experiments with transient EAAT2 blockade with WAY-213,613 during pairings, inducing LTP for t_{STDP}=±200 ms pairings. Insets correspond to the average of 60 EPSCs during baseline and at 1 hour after STDP pairings. Error bars represent the SD (except in panel b and bar graphs: SEM). *: $p<0.05$; ***: $p<0.001$; ns: not significant by unpaired t test, two-tailed (**a,d,f,h**), one-way repeated-measures ANOVA; post hoc Bonferroni-corrected pairwise comparisons (**b**) or one sample t test, two-tailed (**c,e,g,i**).

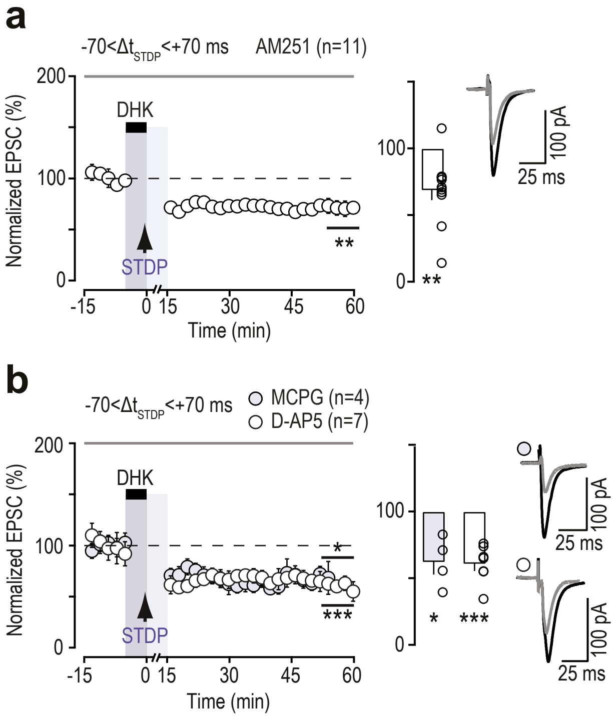

a
-70<Δt_STDP<+70 ms
AM251 (n=11)
DHK
STDP
Normalized EPSC (%)
Time (min)
100 pA
25 ms
b
-70<Δt_STDP<+70 ms
MCPG (n=4)
D-AP5 (n=7)
DHK
STDP
Normalized EPSC (%)
Time (min)
100 pA
25 ms

Supplementary Figure 2. LTD under transient EAAT2 blockade is not CB_1R-, type I/II mGluRs- or NMDAR-mediated

(**a**) LTD under transient EAAT2 blockade for pairings at -70< t_{STDP}<+70 ms was not dependent on CB_1R activation, because AM251 (3 μM) failed to prevent LTD. (**b**) LTD was not mediated by type-I/II mGluR or NMDAR, because MCPG (500 μM) or D-AP5 (50 μM) failed to block LTD.

Insets correspond to the average of 60 EPSCs during baseline and at 1 hour after STDP pairings. Error bars represent the SD (except in bar graphs: SEM). *: $p<0.05$; **:$p<0.01$; ***: $p<0.001$ by one sample t test.

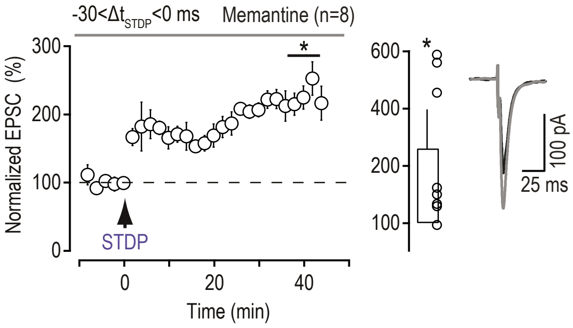

-30<Δt_{STDP}<0 ms
Memantine (n=8)
Normalized EPSC (%)
300
200
100
0
*
STDP
0
20
40
Time (min)
600
400
200
100
*
100 pA
25 ms

Supplementary Figure 3. t-LTP is not dependent on extrasynaptic NMDARs

Memantine (10 μM) did not affect t-LTP for pairings at $-30 < t_{STDP} < 0$ ms in control conditions.

Insets correspond to the average of 60 EPSCs during baseline and at 1 hour after STDP pairings. Error bars represent the SD (except in bar graph: SEM). *: $p<0.05$ by one sample t test.

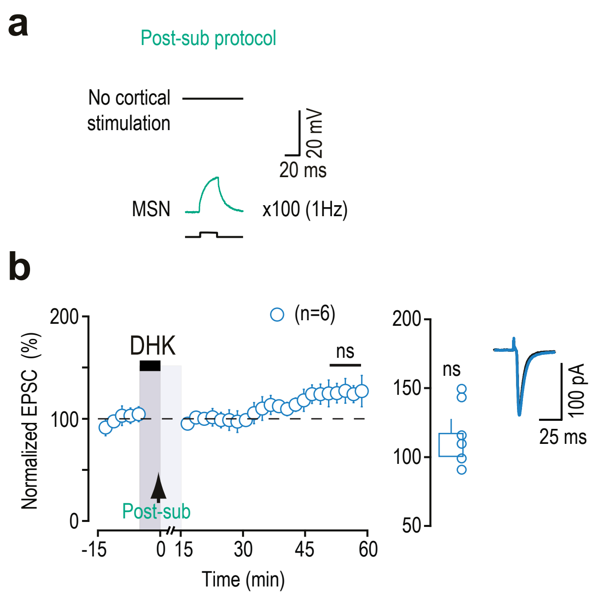
a
Post-sub protocol
No cortical stimulation
20 mV
20 ms
MSN
x100 (1Hz)
b
(n=6)
DHK
ns
Post-sub
Normalized EPSC (%)
200
100
0
-15
0
15
30
45
60
Time (min)
200
150
100
50
ns
100 pA
25 ms

Supplementary Figure 4. Postsynaptic subthreshold activity fails to induce plasticity under EAAT2 blockade

(**a**) Protocol consisting of postsynaptic subthreshold depolarization without paired presynaptic stimulation repeated 100 times at 1 Hz, under EAAT2 blockade. (**b**) This protocol did not induce plasticity.

Insets correspond to the average of 60 EPSCs during baseline and at 1 hour after STDP pairings. Error bars represent the SD (except in bar graph: SEM). ns: not significant by one sample t test.

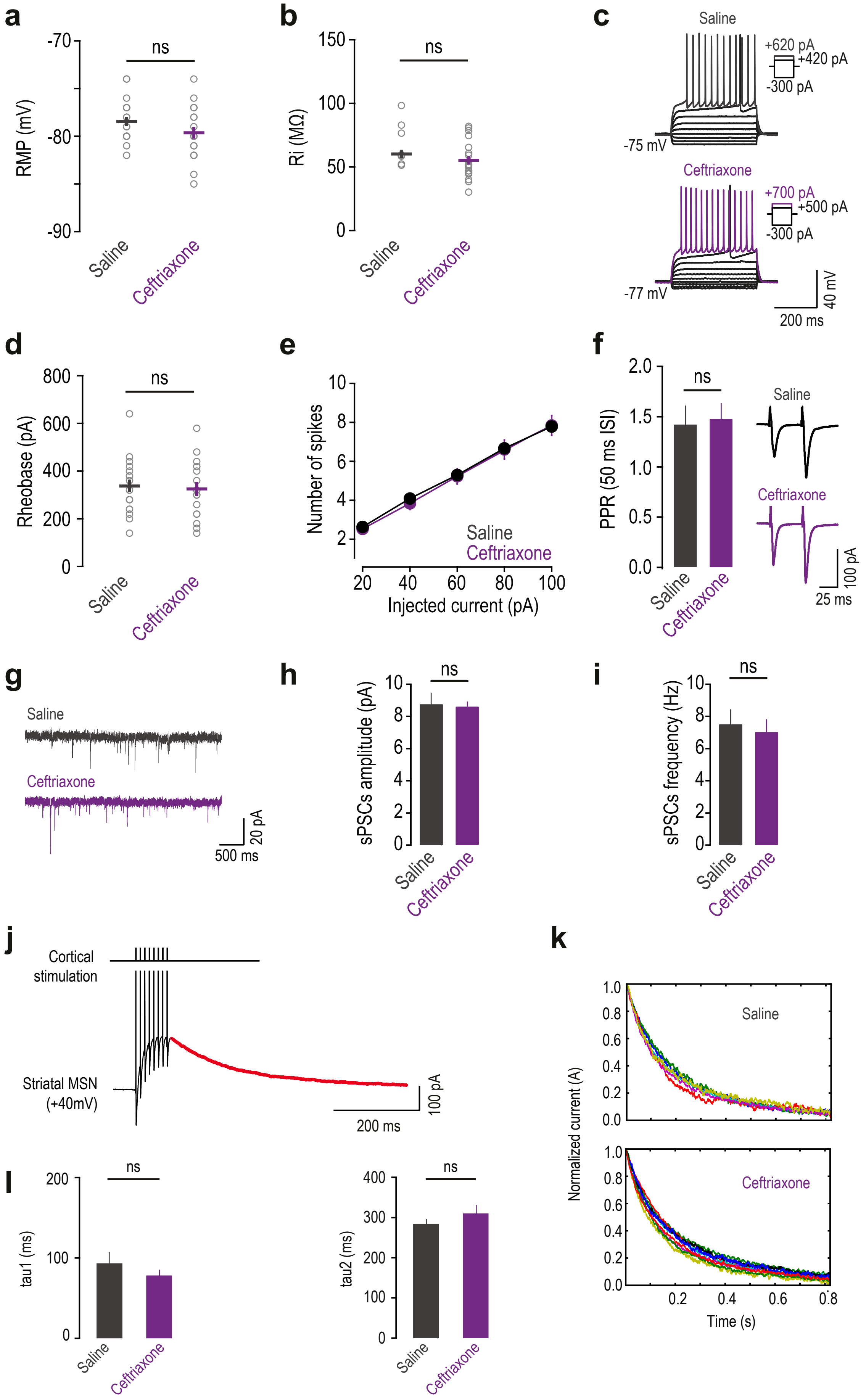
a
RMP (mV)
-70
-80
-90
ns
Saline
Ceftriaxone
b
Ri (MΩ)
150
100
50
0
ns
Saline
Ceftriaxone
c
Saline
+620 pA
+420 pA
-300 pA
-75 mV
Ceftriaxone
+700 pA
+500 pA
-300 pA
-77 mV
40 mV
200 ms
d
Rheobase (pA)
800
600
400
200
0
ns
Saline
Ceftriaxone
e
Number of spikes
10
8
6
4
2
20
40
60
80
100
Injected current (pA)
Saline
Ceftriaxone
f
PPR (50 ms ISI)
2.0
1.5
1.0
0.5
0.0
ns
Saline
Ceftriaxone
Saline
Ceftriaxone
100 pA
25 ms
g
Saline
Ceftriaxone
20 pA
500 ms
h
sPSCs amplitude (pA)
10
8
6
4
2
0
ns
Saline
Ceftriaxone
i
sPSCs frequency (Hz)
10
8
6
4
2
0
ns
Saline
Ceftriaxone
j
Cortical stimulation
Striatal MSN (+40mV)
100 pA
200 ms
k
Normalized current (A)
1.0
0.8
0.6
0.4
0.2
0.0
Saline
Ceftriaxone
0.2
0.4
0.6
0.8
Time (s)
l
tau1 (ms)
200
100
0
ns
Saline
Ceftriaxone
tau2 (ms)
400
300
200
100
0
ns
Saline
Ceftriaxone

Supplementary Figure 5.

MSN properties did not differ between saline- and ceftriaxone-injected rats

(**a**, **b**) The passive electrophysiological properties, RMP (**a**) and Ri (**b**), of MSNs did not differ between saline- and ceftriaxone-injected rats (*n*=20 in both groups). (**c**) Characteristic voltage responses of MSNs from saline- and ceftriaxone-injected rats to a series of 500 ms current pulses. (**d**) The rheobase of MSNs did not differ between saline- and ceftriaxone-injected rats (*n*=20 in both groups). (**e**) Number of elicited spikes plotted as a function of 500 ms current pulses of increasing amplitude in saline- and ceftriaxone-injected rats. No difference was found between the two groups. (**f**) Paired-pulse ratio at 20 Hz induced facilitation did not differ between saline- and ceftriaxone-injected rats (*n*=13 and *n*=16, respectively). (**g**) Traces of sPSCs from saline- and ceftriaxone-injected rats. (**h**, **i**) No difference was found in the amplitude (**h**) and frequency (**i**) of sPSCs between the two groups (*n*=13 and *n*=12, respectively). (**j**) Stimulation protocol for recording NMDAR-EPSCs. Eight presynaptic stimulation pulses were elicited at 100 Hz and neurons were recorded at +40 mV holding voltage. Decay time of NMDAR-EPSCs was analyzed (red trace). (**k**) Normalized currents from saline- and ceftriaxone-treated rats: each color represents a single neuron. (**l**) The fast and slow decay times (tau1 and tau2, respectively) of NMDAR-EPSCs do not show significant difference between saline- and ceftriaxone-injected rats.

Error bars represent the SEM. ns: not significant by unpaired t test, two-tailed.
